# Structure–Property Relationship of Piezoelectric Properties in Zeolitic Imidazolate Frameworks: A Computational Study

**DOI:** 10.1021/acsami.2c13506

**Published:** 2022-11-02

**Authors:** Srinidhi Mula, Lorenzo Donà, Bartolomeo Civalleri, Monique A. van der Veen

**Affiliations:** † Department of Chemical Engineering, Technische Universiteit Delft, Delft2629HZ, The Netherlands; ‡ Dipartimento di Chimica, Università di Torino, Via P. Giuria 7, 10125Torino, Italy

**Keywords:** metal−organic frameworks, DFT, Born effective charge, piezoelectric constant, shear modulus, compliance constant, CdIF-1

## Abstract

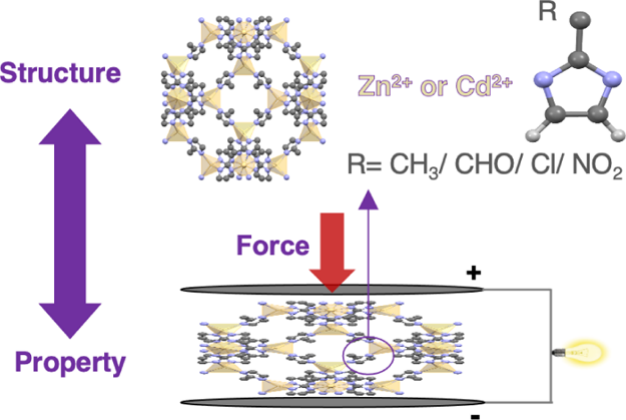

Metal–organic frameworks (MOFs) are a class of nanoporous crystalline materials with very high structural tunability. They possess a very low dielectric permittivity ε_r_ due to their porosity and hence are favorable for piezoelectric energy harvesting. Even though they have huge potential as piezoelectric materials, a detailed analysis and structure–property relationship of the piezoelectric properties in MOFs are lacking so far. This work focuses on a class of cubic non-centrosymmetric MOFs, namely, zeolitic imidazolate frameworks (ZIFs) to rationalize how the variation of different building blocks of the structure, that is, metal node and linker substituents affect the piezoelectric constants. The piezoelectric tensor for the ZIFs is computed from ab initio theoretical methods. From the calculations, we analyze the different contributions to the final piezoelectric constant *d*_14_, namely, the clamped ion (*e*_14_^0^) and the internal strain (*e*_14_^int^) contributions and the mechanical properties. For the studied ZIFs, even though *e*_14_ (*e*_14_^0^ + *e*_14_^int^) is similar for all ZIFs, the resultant piezoelectric coefficient *d*_14_ calculated from piezoelectric constant *e*_14_ and elastic compliance constant *s*_44_ varies significantly among the different structures. It is the largest for CdIF-1 (Cd^2+^ and −CH_3_ linker substituent). This is mainly due to the higher elasticity or flexibility of the framework. Interestingly, the magnitude of *d*_14_ for CdIF-1 is higher than II–VI inorganic piezoelectrics and of a similar magnitude as the quintessential piezoelectric polymer polyvinylidene fluoride.

## 1 Introduction

Energy harvesting is a process where ambient energy in the environment such as mechanical vibrations, light, and heat can be stored and converted into electrical energy. This has been of significant interest to researchers due to its ability to achieve self-powered low-power electronic devices for the Internet of Things and can pave a path to alternate sustainable sources of power. Among the different sources of available energy, kinetic energy in the form of mechanical vibrations is widely available in the environment and various human activities. It can be harvested by piezoelectric materials. The phenomenon of piezoelectricity describes the coupling between the mechanical and electrical properties of materials. This is observed in non-centrosymmetric crystalline structures, where either an electric dipole moment is generated on the application of mechanical stress or a mechanical strain is induced on the application of the electric field.^1^

Piezoelectricity has been found in traditional materials such as inorganic ceramic oxides and organics polymers, among which a high piezoelectric constant was found in lead zirconate titanate (PZT, 360 pC/N) and barium titanate (BaTiO_3_, 191 pC/N) for inorganics, while among soft materials, polyvinylidene fluoride (PVDF) and its copolymers (−40 pC/N) have a high piezoelectric constant. Ceramics and polymers have their own set of advantages and disadvantages as piezoelectric materials, which are summarized and reviewed in the previous literature.^2,3^ Ceramics have strong piezoelectric properties but are stiff and brittle, whereas polymers have mild processing conditions and excellent flexibility, making them suitable for physically flexible electronics, but do not have very high piezoelectric coefficients.^3,4^ Moreover, in contrast to inorganics, the acoustic static impedance of polymers is better matched to that of water and living tissues, making them better equipped to harvest energy in these media.^5^ With the aim of combining the best of both polymer and ceramic piezoelectric components, composite and hybrid materials were fabricated. For example, in recent years, hybrid organic–inorganic perovskite materials emerged as potential candidates with high piezoelectric response close to conventional ceramic oxides. One of the initial works on trimethylchloromethyl ammonium trichloromanganese (TMCM-MnCl_3_) hybrid perovskites (a BaNiO_3_-like structure) shows a piezoresponse *d*_33_ as large as 185 pC/N.^6^ These hybrid perovskites have features from both organic and inorganic materials and have the advantages of being easy to process, lightweight, and structurally tunable. Other recent works on hybrid perovskites show the huge potential of these materials in terms of their piezoelectric constants.^7,8^ Additionally, due to the regulations imposed on the use of hazardous substances such as lead and other heavy metals in electrical and electronic equipment, there has been a need for exploring lead-free environmentally friendly piezoelectrics. Even among the traditional ceramics, lead-free ceramics such as modified potassium sodium niobate (KNN), bismuth sodium titanate (BNT), and other bulk materials were explored as alternatives to PZT.^9,10^ Yet, the disadvantages of ceramics are still relevant for these lead-free materials. Another step toward lead-free piezoelectrics was also achieved by obtaining a large piezoelectric response in metal-free organic perovskites: the *d*_15_ of metal-free MDABCO-NH_4_-X_3_ (with X = Cl, Br, or I) calculated from first-principles calculations show large values of 119, 248, and 178 pC/N respectively.^11^ Like hybrid perovskites, metal–organic frameworks (MOFs) have a major advantage of structural tunability; the building blocks of MOFs, that is, the metal node and connecting organic linker, can be chosen and varied to obtain lead-free piezoelectric structures.

MOFs are materials that consist of metal ions or inorganic clusters connected by organic linkers through directional coordination bonds into a three-dimensional crystalline nanoporous framework. They are distinguished for their permanent porosity and high surface areas. By proper selection of the metal nodes and organic linkers in MOFs, structural control can be achieved to obtain desired properties for the target application. The pore size of MOFs can be changed from several angstroms to nanometers by varying the length of organic linkers in the MOF. Because of these features, MOFs are ideal candidates for applications including but not limited to gas separation and storage, catalysis, and biomedical imaging.^12,13^ In addition to these applications, the second-order nonlinear optical properties via the rational design of non-centrosymmetric MOFs have also been explored previously.^14−16^

So far, only two papers have shown piezoelectric response experimentally in MOFs by quasi-static measurements (measured using a PM200 piezometer): in one *d*_33_ value of 60.10 pC/N was measured for MOFs based on Cd[imazethapyr];^17^ in the other, the *d*_22_ value of Mn-/Co-based homochiral coordination frameworks is measured to be 6.9 pC/N.^18^ There are other works on piezoelectricity in MOFs where electromechanical (EM) response was measured using piezoelectric force microscopy (PFM).^19−21^ Whereas they are worthy to be mentioned, it must be noted that several nonpiezoelectric effects can induce additional contributions to PFM response, thus leading to misinterpretation. Several issues due to the calibration of the microscope and challenges in excluding the contribution of electrostatic and other forces have been discussed in the literature, thus indicating that piezoelectric constants with high precision cannot be measured from PFM in most cases.^22−24^ Recently, also the theoretical prediction of the piezoelectric tensor for three MOFs whose *d*_*ik*_ values range between 2 and 23 pC/N has been reported.^25^

Another factor that is considered important in piezoelectric energy harvesting applications is the dielectric constant of the material, as noted from the figure of merit (FOM)  with units m^2^/N, where ε_33_^*T*^ is the relative permittivity (or dielectric constant) of the material at constant stress (*T*).^26^ In relation to the dielectric constant of MOFs, because of their inherent permanent porosity, they generally have low dielectric constants (ε_r_) ranging between 1.3 to 6.^27−32^ Note that the computationally obtained dielectric constants are static dielectric constants and that the experimental determination via dielectric spectroscopy is often done on air–MOF composites (pellets), both leading to some underestimation of the dielectric constant. Yet, the reported values are very low, and, for example, a study where the determination of the dielectric constant of ZIF-8 was performed via ellipsometry on a continuous film, a method not prone to underestimation, reports a low value of 2.3.^27^ These values are very low compared to the dielectric constants of BaTiO_3_, PZT, and PVDF and copolymers shown previously. If a high EM response can be achieved by tuning the topology and building blocks of the material, combined with such low dielectric constants, MOFs present an interesting class of materials to explore for efficient energy harvesters.

**Figure 1:**
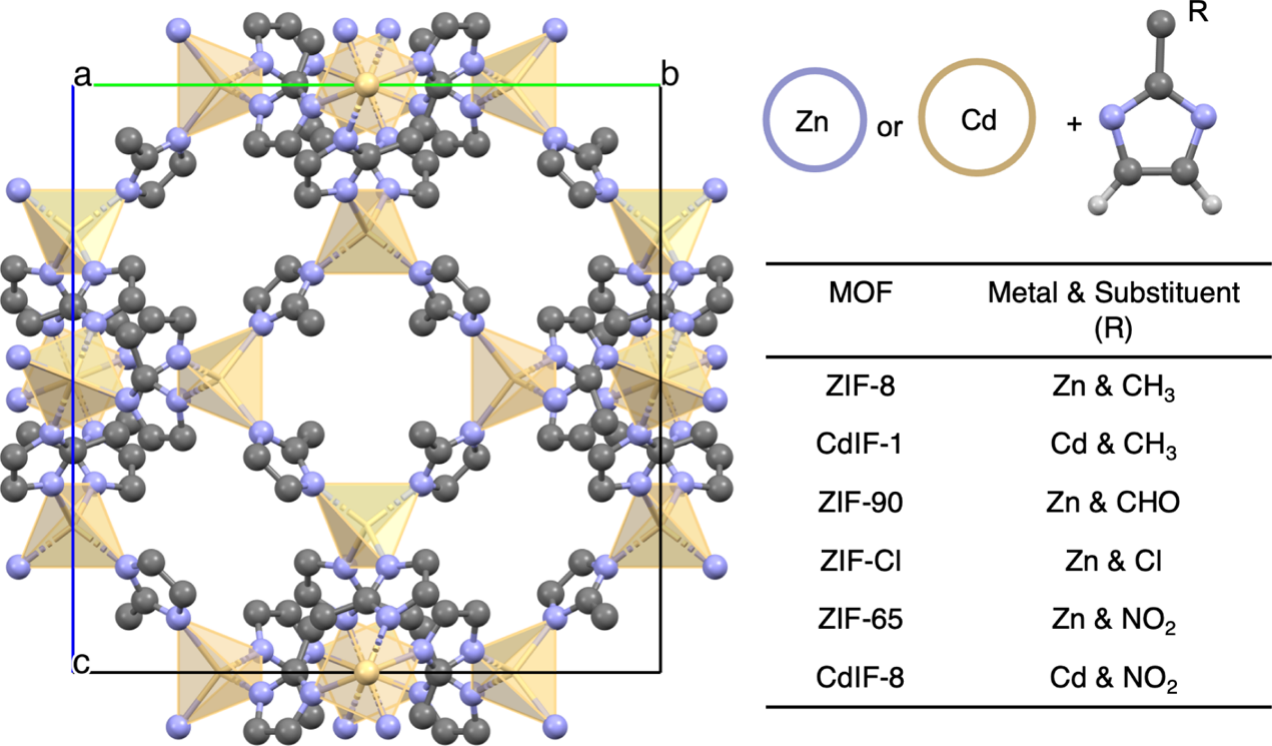
Representation of the unit cell of ZIFs (left) and name of the ZIF along with their metal node and the substituent on the imidazolate linker included for different ZIFs in this work.

The experimental and computed data for MOFs are thus quite promising in terms of the piezoresponse. However, the MOFs studied differ widely in their building units and structure. Thus, it is hard to derive a structure–property relationship that will guide the design of better-performing piezoelectric MOFs. In the present work, we aim at filling this gap. To this purpose, we chose to focus on zeolitic imidazolate frameworks (ZIFs), a subfamily of MOFs consisting of M–Im–M, where M is the metal cation (i.e., Zn, Cd, or Co) and Im is the imidazolate organic ligand and its derivatives. The reference to zeolites in the naming of ZIFs relates to their topological similarity. Zeolites consist of tetrahedrally bonded Si^4+^ (or Al^3+^) atoms connected by oxygen atoms, forming Si/Al–O–Si/Al angles of 145°. Similarly, in ZIFs, the metal (M^2+^) cations are tetrahedrally coordinated to nitrogen at 1,3-positions of the organics imidazolate linkers, leading to a M–Im–M angle of 145°. This leads to this class of metal–organic frameworks, forming the same net topologies as zeolites. Due to the organic linkers and metal–organic bonds, ZIFs generally have higher flexibility compared to zeolites. ZIFs are also known to have excellent thermal (can withstand up to 550 °C) and chemical stability.^33^ In this work, we specifically focus on sodalite (sod) ZIFs with Zn or Cd metal nodes and R-substituted imidazolate linkers (R = CH_3_, Cl, CHO, NO_2_) as shown in Figure 1. Examined ZIFs belong to non-centrosymmetric space groups (i.e., *I*4̅3*m*) with a large degree of tunability while keeping the same topology of the prototypical ZIF, that is, ZIF-8 (with Zn and R = CH_3_). We calculated the pore size, which is the diameter of the largest sphere that will fit into the ZIF framework. The largest pore size for Zn–ZIFs in this work (i.e., ZIF-8, ZIF-90, ZIF-Cl, and ZIF-65) ranges between 11.5 and 12 Å.^33^ For Cd–ZIFs (i.e., CdIF-1 and CdIF-8), the pore size varies between 14.2 and 15.2 Å,^34^ which is slightly higher than that of Zn–ZIFs. This is due to the slightly longer Cd–N bond compared to Zn–N. Such high porosity should contribute to a lower dielectric constant favorable for piezoelectric energy harvesting. Indeed the total porosity for ZIFs in this work varies between 48 and 60% of the volume of the unit cell. Notably, from an application point-of-view, among the studied ZIFs, ZIF-8 was already shown to have a very low dielectric constant of 2.3–2.45 with a low loss factor,^27,35^ and can be grown into thin films showing good processibility. Overall, the selected ZIFs are ideal candidates for studying structure–property relationships.

In this paper, we present a systematic computational study on how piezoelectric response varies with changes in building units of ZIFs. For this, we change the metal node (Zn, Cd) and substituent on the imidazolate linker in ZIFs. Piezoelectric coefficients for the investigated ZIFs were calculated using different density functional theory (DFT) methods. To understand the contributing factors of piezoelectric response in ZIFs, we dissect the different contributions of piezoresponse to a mechanical strain into clamped ions, internal strain contributions, and Born effective charges (BECs). We then discuss the results of mechanical properties (specifically the compliance constant *s*_44_) and the piezoelectric coefficient for the different ZIFs. For this class of materials, we show that the mechanical properties have a dominant contribution to the piezoelectric constant *d* than the contribution from the piezoresponse *e*. In the last part of the paper, we compare these results of ZIFs with some existing Zn/Cd-based inorganic and organic piezoelectrics.

## 2 Theory

Piezoelectricity is mathematically described in the IEEE standard for piezoelectricity^36^ by a set of four constitutive equations that describe the response of a piezoelectric material to a mechanical load (stress/strain) and electric fields. The relevant set of equations involving the mechanical and electrical variables and their units are discussed in detail in Supporting Information Section 1. The piezoelectric tensors with units [C/m^2^] and [pC/N], respectively, and of importance in piezoelectric energy harvesting applications to store as energy is the value of *d* which effectively links the applied stress (*T*) to the electric displacement (*D*). We use the Voigt notation for the third-order piezoelectric tensors (*e*_*ikl*_ and *d*_*kij*_) and fourth-order compliance tensor (*s*_*ijkl*_^E^) where the indices are given in a compressed matrix notation instead of the tensor notation. Hence, they can be rewritten as *e*_*iq*_, *d*_*ip*_, and *s*_*qp*_^E^. The piezoelectric constant *d*_*ip*_ can be computed from the piezoelectric constant *e* and the elastic compliance constant *s* using *d*_*ip*_ = *e*_*iq*_*s*_*qp*_^E^. Computationally, the piezoelectric constant *e*_*iq*_ is calculated as the first derivative of the magnitude of the polarization *P* induced by strain .

The piezoelectric tensor *e* can be further separated into two parts: clamped ion (*e*_14_^0^) and internal strain (*e*_*ik*_^int^) contributions. This is according to a well-known scheme used for many inorganic piezoelectrics to understand the origin of piezoelectricity in the material.^37−39^ As indicated in the work by Catti et al.^40^ for a general case, piezoelectric constant *e* is shown in eq 1.
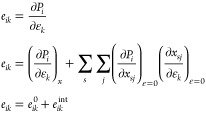
1

Essentially, the clamped ion contribution (*e*_*ik*_^0^) is the change in polarization *P*_*i*_ due to the reorganization of electron density with external strain ε_*k*_, while the fractional coordinates *x* are kept constant in the strained unit cell, that is, . The internal strain contribution (*e*_*ik*_^int^) provides the change in polarization *P*_*i*_ where the atoms are allowed to relax in the strained unit cell in response to the strain. This means that the internal strain contribution and the clamped ion contribution are typically opposite in sign. For the internal strain contribution, *e*_*ik*_^int^, we sum over all the atoms “*s*” in the unit cell along three directions “*j*” (1, 2, and 3). In this contribution, the change in polarization *P*_*i*_ with fractional coordinate *x*_*sj*_ = 0, , is multiplied by the change in fractional coordinates *x*_*sj*_ with the lattice strain, also termed the relaxation coefficient . Essentially, the internal strain contribution refers to the inner deformation of the crystal structure to keep the energy minimum. Moreover, , is related to, *Z*_*s*,ij_^*^, that is, the BEC.^40^ The BEC is a dynamical charge introduced by Max Born and Maria Göppert Mayer. It refers to a change in polarization induced by an atomic displacement under the condition of zero macroscopic electric field.^41^

Hence, for a cubic system, such as the investigated ZIFs, the internal strain contribution can be written in terms of BECs, as indicated in eq 2.
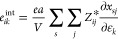
2where *a* is the lattice parameter and *V* is the unit-cell volume of the unstrained structure. Thus, the internal strain contribution is obtained by summing the product of BECs, a second-order tensor (BECs *Z*_*s*,ij_^*^) and respective relaxation coefficient . From eqs 1 and 2, fully understanding the *e*_*ik*_ in any piezoelectric material requires the determination of three quantities (a) *e*_*ik*_^0^ clamped ion contribution, (b) *Z*_*s*,ij_^*^ BECs, and (c) , the relaxation coefficient, for all atoms in the unit cell. Note the relevance of the BECs: the high piezoelectric response of the best-performing ceramics is due to very high anomalous dynamical BECs of their transition metals (M) (vide infra). Further research on these dynamical charges in ceramic oxides indicated the mixed covalent and ionic character of the M–O bonds. Dynamical changes in the hybridization of these bonds with atomic displacement results in charge reorganization, which is observed as the BEC of atoms.^41^

The piezoelectric tensors for the investigated ZIF space group *I*4̅3*m* have only one independent piezoelectric constant: *e*_14_ and *d*_14_. Specifically, for these space groups, eqs 1 and 2 can be rewritten as shown in eq 3. The relation between piezoelectric constants *e* and *d* via compliance constant *s* is shown in eq 4.
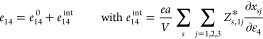
3
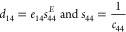
4where *c*_44_ is the shear elastic constant and *s*_44_ is the compliance constant.

## 3 Methods

All the calculations reported in this paper are performed using the ab initio periodic code CRYSTAL17^42^ based on the atom-centered Gaussian basis set. Triple zeta basis sets with polarization functions for all atoms^43−47^ and Grimme D3 dispersions corrections were used.^48^ For metal atoms, Zn and Cd extra polarization functions were included.^49,50^ We adopted five DFT functionals at the generalized gradient approximation (GGA) level of theory, namely, hybrid functionals B3LYP and PBE0^51,52^ with 20 and 25% of HF exchange, meta GGA functional M06L^53^ with 0% HF exchange, and meta hybrid functionals M06 and M062X^54^ which include 27 and 54% HF exchange, respectively. First, we performed a full relaxation (cell parameters and atomic positions) of the ZIF structures with all DFT functionals. Lattice parameters of structures taken from experimentally determined parameters reported in the CSD database^55^ were used for structural relaxation. The deviation between experimental and simulated lattice parameters for all functionals is always below ±2%, as shown in Table S1. Using the optimized structure as starting geometry, full piezoelectric and elastic tensors were calculated using the numerical approach based on the geometry optimization of atomic positions at strained lattice configurations. Piezoelectric constant *e* is evaluated using the Berry phase approach in the modern theory of polarization^56−58^ as a first derivative of the Berry phase^59^ with respect to strain.^60^ This is done in CRYSTAL17 by applying finite strains to the lattice as finite differences of polarization at strained configurations. The elastic or compliance constants *c* or *s* are expressed as second derivatives of energy with respect to pairs of strains, wherein CRYSTAL17, the first derivative, is computed analytically, while the second derivative is evaluated as numerical finite differences between strained structures described in the work by Erba et al.^61^ The other piezoelectric constant *d* is evaluated from the relation between *e* and *c* or *s* shown in eq 4. ZIF structures were subjected to a finite strain of ±1.5% for the numerical differentiation of the analytic cell gradients. We obtained piezoelectric and mechanical properties for all five DFT functionals; however, our most elaborate discussion in the main paper will involve the results using B3LYP, which will be motivated later in the text. The raw data files with the representative input files and output files of the calculations done in this work can also be accessed by others at 4TU.ResearchData.^76^

## 4 Results and Discussion

**Figure 2:**
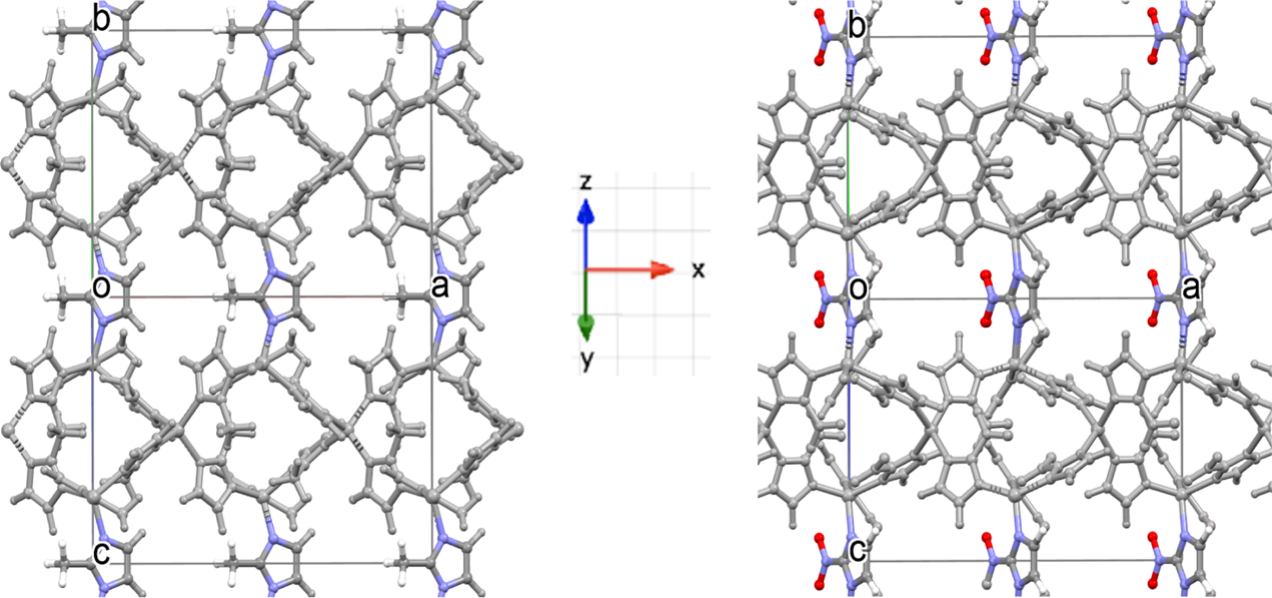
Geometry-optimized equilibrium structures of ZIF-8 (left) and ZIF-65 (right) shown along the (011) plane showcasing the linkers with their C2 axis aligned dipolarly along “*x*”.

In this study, we consider six different ZIFs with Zn/Cd as the metal node and substituted imidazolate as the linker, namely, ZIF-8, CdIF-1, ZIF-90, ZIF-Cl, ZIF-65, and CdIF-8. The four different substituents of imidazolate considered are CH_3_, CHO, Cl, and NO_2_. Figure 2 shows the equilibrium structure of methyl and nitro-substituted ZIFs, where part of the linkers are oriented along the dipolar direction *x* (denoted 1 in Voigt’s notation), the direction along which we model the change in the electric response while applying a shear deformation along the depicted *y* and *z* directions (i.e., 2 and 3 directions denoted as 4 in Voigt’s notation). This is also the same for other studied ZIFs.

We first present the detailed outcomes of *e*_14_ for all ZIFs in terms of the clamped ion (*e*_14_^0^) and internal strain contributions (*e*_14_^int^). Later, we will discuss the mechanical properties, that is, *s*_44_ and resultant *d*_14_. The analysis of piezoelectric constant “*e*” by separating into the clamped ion and internal strain contributions is also applicable to perovskite materials and has been done previously for inorganic piezoelectrics.^37,39,40,62^ We used a series of different functionals (B3LYP, PBE0, M06, M06L, and M062X) and the lattice parameters of geometry-optimized structures with all functionals are very close to experimental values with a deviation <2% as shown in Table S1 in the Supporting Information. However, the variation of piezoelectric and mechanical properties with the DFT functional is small for some ZIFs, while it is large for others. Here, in the main paper, we draw only conclusions on variations in properties between different ZIFs when these variations between ZIFs are larger than the variation between functionals for the same ZIF. In the main paper, we elaborate and discuss the results obtained with B3LYP in depth as this functional has shown the best correspondence with experimental values for the elastic properties of ZIF-8.^63^

**Figure 3:**
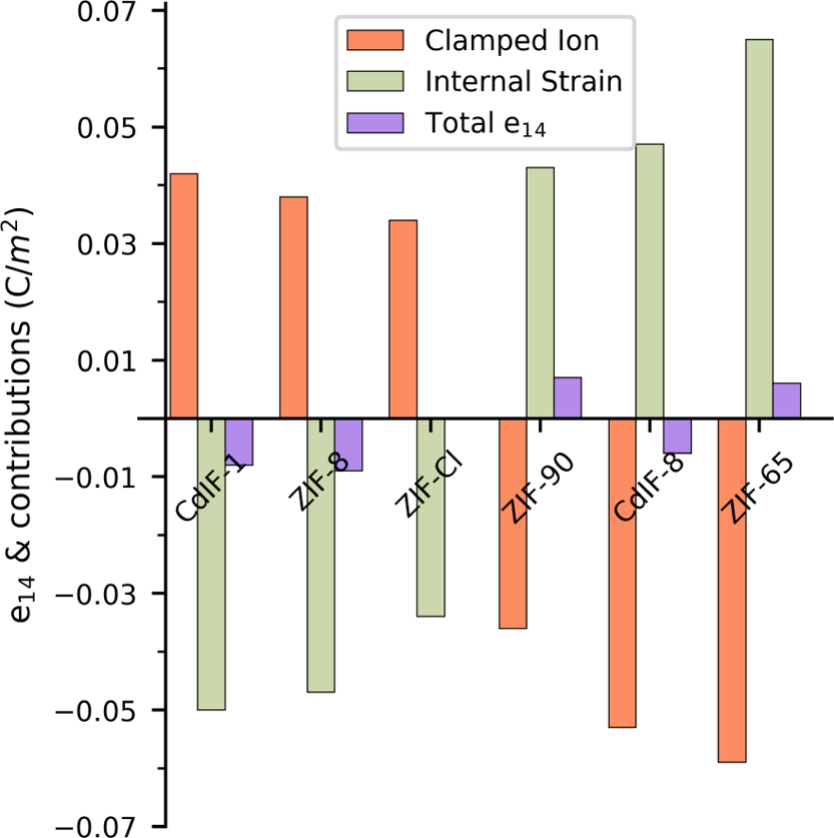
Bar graph showing the total piezoelectric constant (*e*_14_) and its components, the clamped ion (*e*_14_^0^), and internal strain contributions (*e*_int_^0^), for the ZIFs considered ZIFs, modeled with the B3LYP functional.

Figure 3 shows the total *e*_14_, the clamped ion contribution (*e*_14_^0^), and internal strain contribution (*e*_14_^int^) for all ZIFs. As seen in Figure 3, the sum of both contributions *e*_14_ has much lower absolute values due to the expected opposing sign of the two contributions, with the absolute magnitude of both contributions being very similar. Due to the small magnitude of *e*_14_ compared to the variation in magnitude between different DFT functionals, it is most meaningful to discuss changes in *e*_14_^0^ and *e*_14_^int^ between the different ZIF structures, rather than the differences in the resultant *e*_14_.

### 4.1 Clamped Ion Contribution (*e*_14_^0^)

**Figure 4:**
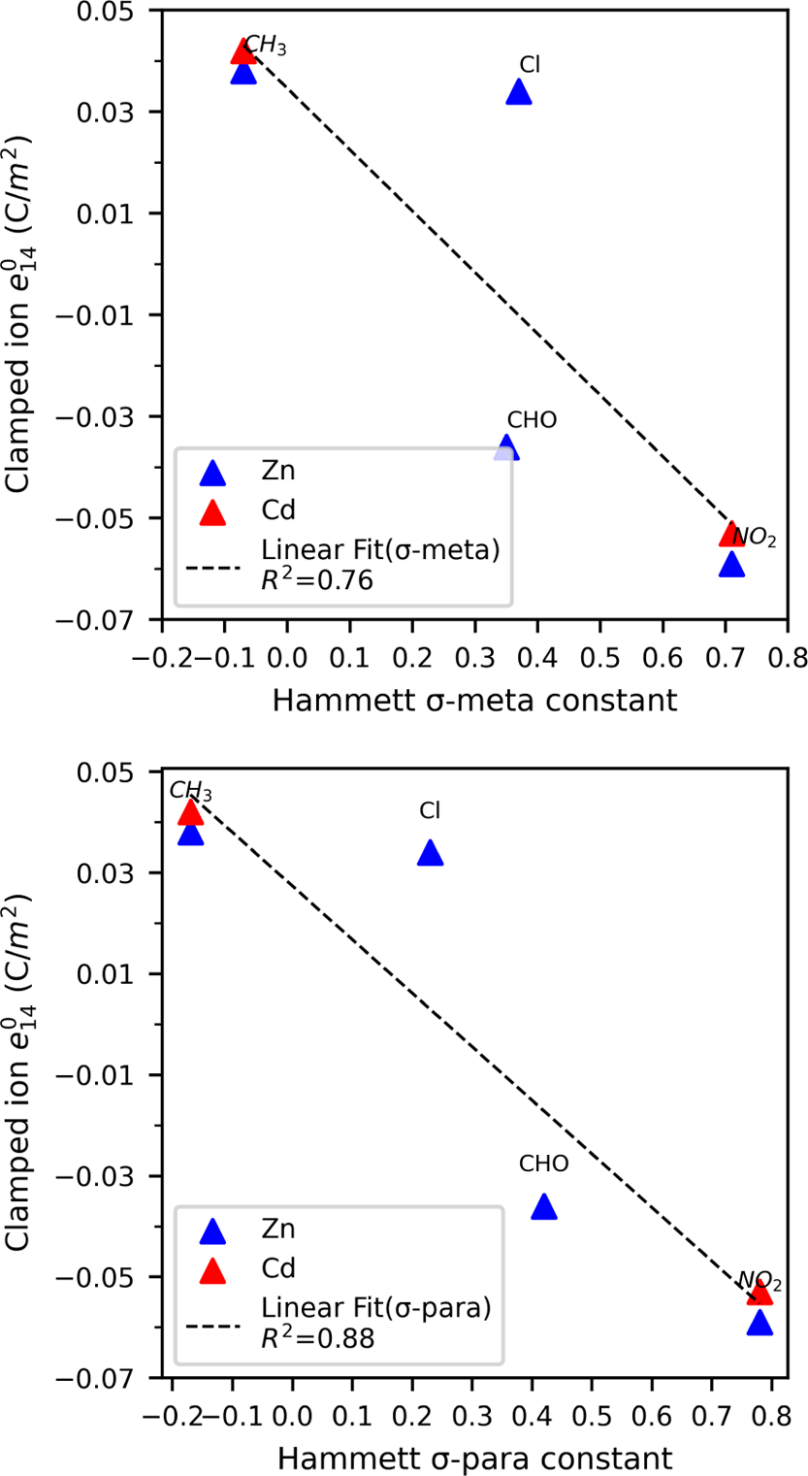
Correlation between clamped ion contribution *e*_14_^0^ and Hammett σ meta and para constants for four different substituents of imidazolate.

The sign of *e*_14_^0^ changes from positive for −CH_3_ ZIFs to negative *e*_14_^0^ for −NO_2_ ZIFs (Figure 3); in effect, this is a manifestation of the dipole moment of the linker changing sign going from an electron-donating to an electron-withdrawing side group. As the clamped ion contribution is a purely electronic contribution that indicates the effect of redistribution of electron density upon strain, it is informative to compare trends in the clamped ion contribution with the well-established and easily accessible parameter Hammett σ constant. It can be considered a measure of the electronic nature of the linker substituents, with a more positive Hammett constant for electron-withdrawing substituent groups, and a more negative one for the electron-donating ones. The Hammett σ constant has been published in the literature for substituted phenyl systems based on meta or para substitution as σ meta or σ para constants, respectively.^64,65^ It was also used for other chemically distinct systems such as the p*K*_a_ values of imidazole derivatives and electronic, spectroscopic properties of pyrazole derivatives.^66,67^Figure 4 shows a plot of the clamped ion *e*_14_^0^ of ZIFs with Zn/Cd as the metal node and σ meta and σ para constants. Linear fit in the plot shows a good correlation with the coefficient of determination *R*^2^ = 0.76 with σ meta and *R*^2^ = 0.88 with σ para Hammett constants; hence, the electronic nature as captured in the Hammett constant is a good approximation to describe the effect of the linker substituent to *e*_14_^0^ in terms of changing the dipole moment of the linker. As can be seen in Figure 4, the value of *e*_14_^0^ is hardly affected by the choice of metal ion (Zn^2+^ vs Cd^2+^ for −CH_3_ and −NO_2_ substituents).

### 4.2 Internal Strain Contribution (*e*_14_^int^)

For all studied ZIFs, the internal strain contribution is opposite in sign and nearly equal in magnitude to the clamped ion contribution (*e*_14_^0^). As ZIFs are relatively soft materials, it is indeed expected that the nuclei relax (internal strain contribution) in a manner to counter the polarization created via the clamped ion contribution. As a result, the final *e*_14_ is much smaller for all ZIFs when compared to some inorganics such as Zn and Cd oxides and sulfides.^68,69^ As shown in eq 3, the internal strain *e*_14_^int^ is a product of the BECs *Z*_*s*,1j_^*^ and the relaxation coefficient , which depends on the change in positions of the atoms during strain. Concerning the latter, we look at changes due to external shear in all bond lengths and angles in the structure. The largest variation above a cutoff of 0.05% for the relative change in bond lengths and absolute change of 1° for angles in response to an external shear deformation of 3% is considered and shown in Table S2 in the Supporting Information. For all ZIFs, we see that the highest change in bond length occurs in the metal–N bonds ranging from 0.24% for ZIF-65 to 0.06% for ZIF-8. The variation in the bond lengths for all ZIFs being less than 0.24% indicates that bond lengths play a minor role in the shear deformation. Among the angles, the maximum variation for each ZIF constitutes that of the N–metal–N bond angle (from 2.90 to 3.48°), followed by the variation of the metal–N–C bond angle (from 0.69 to 1.44°) (see Table S2). This indicates that the flexibility is centered around the position of the linkers relative to the Zn^2+^ or Cd^2+^ ions and that the organic linker is comparatively rigid. Additionally, the structural changes observed are mainly due to bond angle changes around the metal tetrahedra, while the changes in bond lengths are minimal. The other factors that influence *e*_14_^int^ are the BECs, Z* which are second-rank tensors and can be represented in a matrix form in Voigt notation as
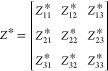


**Figure 5:**
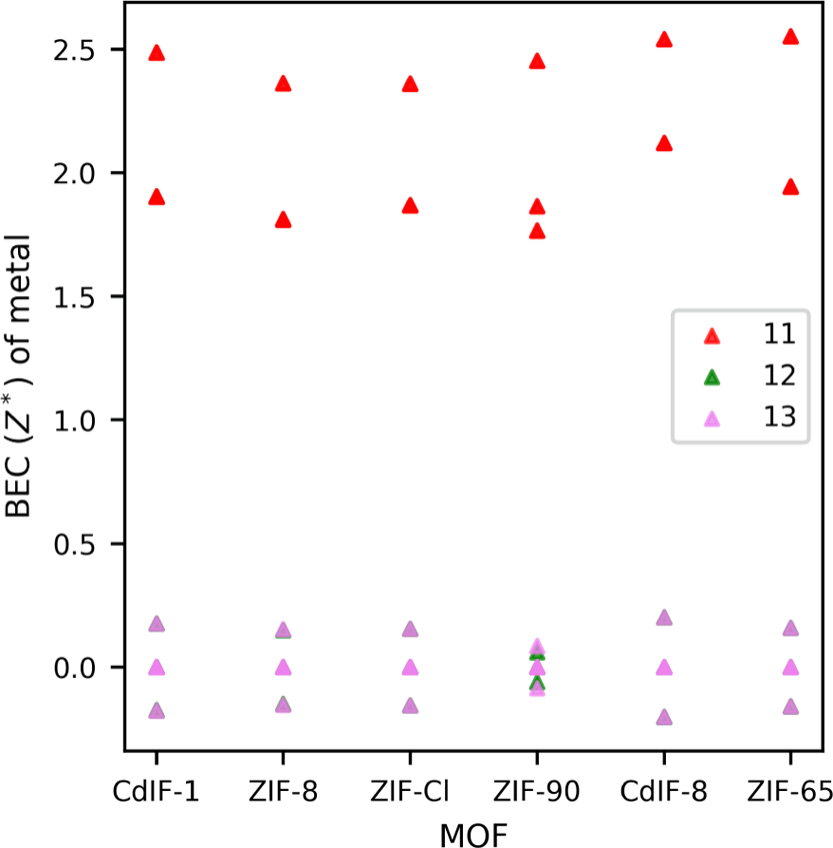
BEC of all metal nodes for the six ZIFs. BEC in relevant directions 11, 12, and 13 is shown with different colored markers.

**Figure 6:**
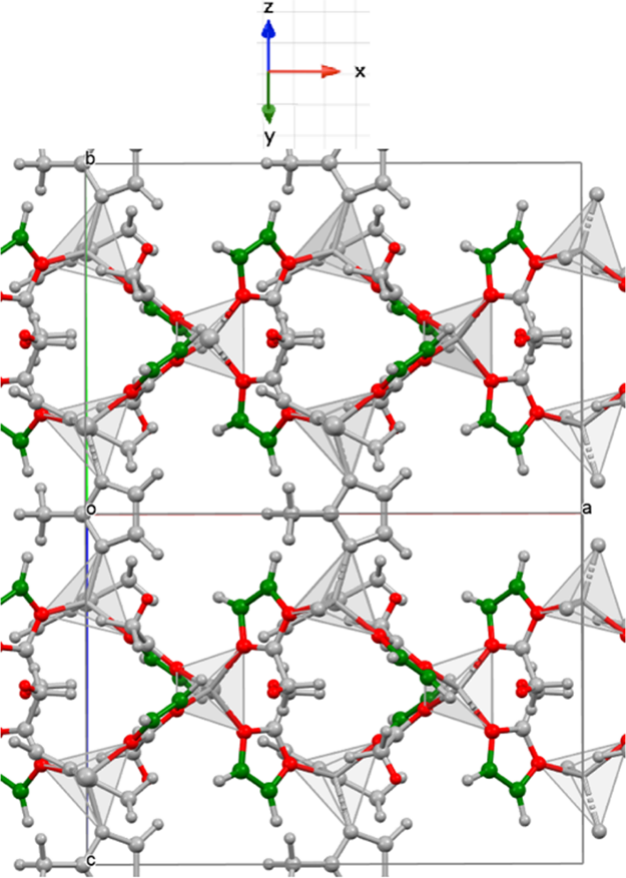
ZIF structure highlighting the atoms with contribution “*A*” =  greater than 5% of |*e*_14_^int^| for both ZIF8 and CdIF-1. The positive contribution of “*A*” is indicated in green color and negative contribution in red color.

In inorganics such as BaTiO_3_, anomalously high BECs are responsible for superior piezoelectric constants, where the BEC is much higher than the nominal charge of the atom (e.g., a BEC (*Z*_Ti_^*^) of +6.7 for Ti with a nominal charge of +4 in BaTiO_3_).^41^ Hence, we analyzed the magnitude of BECs of all atoms in the ZIF structures. For all ZIFs, the acoustic sum rule (i.e., ∑_*s*_*Z*_*s*,ij_^*^ = 0 meaning charge neutrality in all directions) is satisfied, indicating good convergence of our calculations within an accuracy of 0.07 charge units. In eq 3, BEC *Z*_*s*,1j_^*^ with *j* = 1, 2, 3 are the relevant elements of the BEC tensor that contribute to *e*_14_^int^. Figure 5 shows a scatter plot of *Z*_Zn/Cd,11_^*^, *Z*_Zn/Cd,12_^*^, and *Z*_*Z*n/Cd,13_^*^ of all metal atoms Zn and Cd in the unit cell for all six ZIFs in this work. We see that *Z*_Zn/Cd,11_^*^ > *Z*_Zn/Cd,12_^*^,*Z*_Zn/Cd,13_^*^ and values of *Z*_Zn/Cd,11_^*^ are around +2.0 to +2.5. Thus, the overall BEC of the metal node in these ZIFs is close to their nominal charge of +2.0 and not anomalous. The influence of the linker substituent on the BEC of the metal node is negligible, with a variation of around ±0.2. The variation of the BEC of the organic atoms in discussed in the Supporting Information in Section 4.2. Overall, metal atoms have the highest BEC among all atoms in the ZIF system. In the case of the organic part of ZIFs, there is a lot of variation in the BECs depending on the displacement direction, and they mostly range from +1 to −1 indicating the covalent bond nature between them. None of the magnitudes of BECs are exceptionally high, which is one of the reasons why the internal strain component of *e*_14_, that is, *e*_14_^int^, is not high enough to overcome the clamped ion component *e*_14_^0^ significantly, as does happen for the best-performing inorganic piezoelectrics (such as PZT and BaTiO_3_). As *e*_14_^int^ is defined as the product of the BECs and relaxation coefficients (eq 3), we now identify the atoms which have the highest magnitude for this product “*A*” =  for all ZIFs. This product was calculated numerically from the DFT-calculated BEC tensor and change in the positions of atoms obtained from unstrained and strained structures. For each ZIF, a cutoff value of 5% of |*e*_14_^int^| was chosen to consider the product “*A*” =  for all atoms. This value is ∼0.002 to 0.003 C/m^2^ depending on the ZIF. All atoms with absolute contributions greater than this value are highlighted in Figure 6 (for ZIF-8 and CdIF-1) and Figure S6 in the Supporting Information (for ZIF-90, ZIF-Cl, ZIF-65, and CdIF-8), where green indicates a positive contribution and red indicates a negative contribution to internal strain contribution *e*_14_^int^. Since the *e*_14_^int^ refers to polarization along *x*(1) when a shear strain is applied along *yz*(4), this orientation to show the (011) plane was chosen in Figure 6. For the linkers aligned with their C2-axis along the *x*-direction, these are dipolarly aligned. Note, however, that the other linkers are oriented in a manner that diminished the overall polarization caused by the linkers.

For ZIF-8 and CdIF-1, we see a similarity in the atoms that have a higher magnitude for “*A*”; see Figure 6. We see that, overall, the organic linker, which as a mostly rigid unit is tilted with respect to the M^2+^ tetrahedra (vide infra), contributes most to the internal strain piezoelectric coefficient *e*_14_^int^. The larger relocation of the organic atoms  trumps the contribution of the larger BECs of the inorganic atoms (*Z*_*s*,1j_^*^). Most studied ZIF structures show a similar behavior, except for aldehyde and the nitro-substituted ZIFs (ZIF-90, ZIF-65, and CdIF-8): for these structures also, the inorganic atoms show an “*A*” value above the cutoff.

### 4.3 Elastic Compliance Constant (*s*_44_) and Piezoelectric Constant (*d*_14_)

The piezoelectric coefficient that determines the performance in piezoelectric devices is *d*_14_, which we can be derived from *e*_14_ and the elastic compliance constant *s*_44_ (see eq 4). To be specific, *d* is one of the factors responsible for high FOM in energy harvesting applications. As shown in theoretical and experimental work on shear modulus and mechanical properties of ZIF-8 by Tan et al.,^63^ the theoretical estimation of elastic constants depends on the choice of exchange–correlation functionals and dispersion corrections. We calculated *c*_44_ and *s*_44_ with different DFT functionals shown in Figure S7 in the Supporting Information and the ratio of the standard deviation to the average varies from 0.1 to 18%, except for CdIF-1 and CdIF-8 with a deviation of 30%. Although *c*_44_ and *s*_44_ vary with the choice of DFT functionals for each ZIF, variation between different ZIFs is consistent in most cases. As shown in Figure S10 in Supporting Information, correspondingly there is a lot of variation of *d*_14_ with the choice of DFT functional, due to the variability of both *e*_14_ and *s*_44_ with choice of functional. However, the variation between different ZIF structures (the trend) is mostly consistent. Since the B3LYP functional with and without dispersion corrections estimates reliable values close to experimentally measured mechanical properties at 295 K in ZIF-8,^63^ we will discuss and compare the results of *s*_44_ and *d*_14_ with B3LYP for all ZIFs in the main paper. Moreover, we will only point out differences between structures when this difference is larger than the variation of the property with the functional. *c*_44_ was previously calculated by DFT for ZIF-90 and ZIF-Cl and values are 2.502 and 3.578 GPa respectively.^70^ Despite having different values for *c*_44_ in comparison to the previous work, the values in our work computed with all the five different DFT functionals are in the same range, that is, 0.75–1.18 GPa and 0.81–0.91 GPa for ZIF-90 and ZIF-Cl, respectively (see Figure S7a).

**Figure 7:**
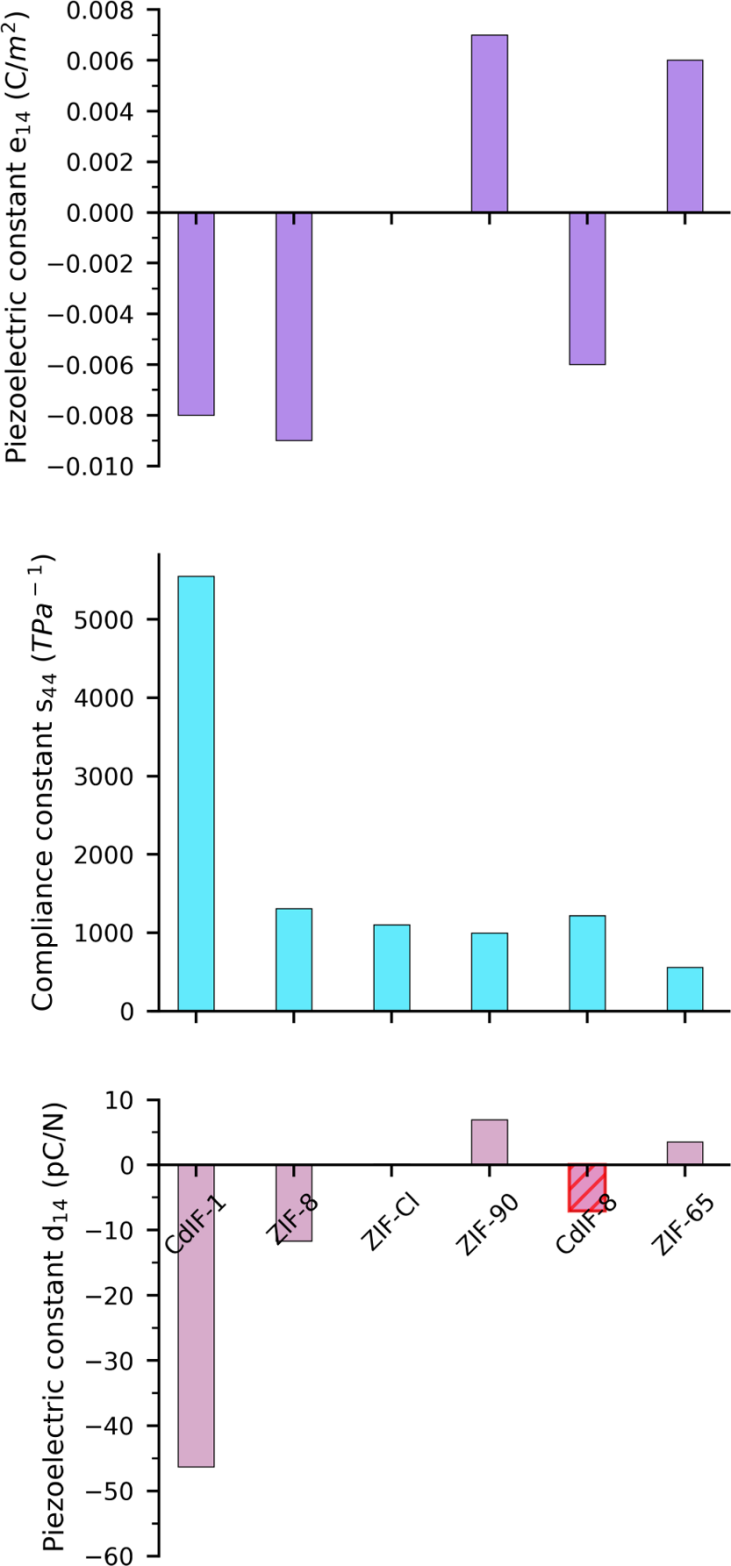
Bar graphs showing *e*_14_, *s*_44_, and *d*_14_ for all six ZIFs with the B3LYP functional (*d*_14_ for CdIF-8 marked in graph, is unreliable due to huge variation in the DFT functional).

Mechanical properties of the investigated ZIFs indicate a change in the flexibility of the framework with a metal node and linker substituent. High *s*_44_ (or low *c*_44_) means more flexibility of the framework, while low *s*_44_ (high *c*_44_) indicates stiffer mechanical properties. As seen in Figure 7, *s*_44_ is the highest for CdIF-1 with Cd and −CH_3_ as substituents and lowest for ZIF-65. Overall, we see that for the same substituent, *s*_44_ increases significantly going from Zn^2+^ to Cd^2+^ and that, overall, −CH_3_ is the substituent leading to the most flexibility and −NO_2_ to the least. Compliance constant *s*_44_ of ZIF-90 and ZIF-Cl range in between −CH_3_ and −NO_2_ substituent Zn–ZIFs. We hypothesize the cause for −NO_2_ ZIFs being stiffer than −CH_3_ ZIFs, although the weaker Zn–N bond in the former is due to the increased steric interactions of the larger −NO_2_ groups. For example, as shown in Figure S11 in the Supporting Information, the oxygens of the −NO_2_ group in adjacent linkers are closer than the hydrogens of the −CH_3_ group (3.095 vs 3.297 Å where van der Waals radii of O and H are 1.5 and 1.2 Å, respectively^71^), due to which there is more resistance during shear, resulting in higher *c*_44_/lower *s*_44_. It is clear that Cd^2+^ ZIFs are more flexible with regard to the shear modulus than Zn^2+^ ZIFs. This could be due to the longer Cd–N bond distance of 2.2 Å compared to the Zn–N bond distance of 2.0 Å.

In the end, looking at the piezoelectric constant *d*_14_, it is the highest in magnitude for CdIF-1 compared to other ZIFs for all DFT functionals used in this work. Since *e*_14_ has the same order of magnitude for all ZIFs, flexibility is the key contributing factor responsible for higher *d*_14_ of CdIF-1. The value of *d*_14_ for CdIF-1 is in the range of −38 to −46 pC/N (except for the M06L functional; it is −94 pC/N; see Figure S8 in the Supporting Information). After CdIF-1, ZIF-8 seems to have *d*_14_ values in the range of −9 to −14 pC/N. For ZIF-90, ZIF-Cl, and ZIF-65, due to huge variation in *d*_14_ with the DFT functional, it is difficult to conclude a trend, but the magnitudes of *d*_14_ are in the same range for these three MOFs, that is, <±8 pC/N. *d*_14_ for CdIF-8 (marked in Figure 7) varies between 2 and 50 pC/N due to a large variation in both *e*_14_ and *s*_44_ with the functional, making it difficult to conclude about *d*_14_ in CdIF-8.

## 5 Comparison with Existing Inorganic and Organic Piezoelectrics

### 5.1 Piezoelectric Properties

Here, we compare the results of piezoelectric and mechanical properties of ZIFs in this work with some existing Zn/Cd inorganic and widely known organic piezoelectrics such as PVDF and its copolymers. Table 1 lists the compliance constants (*s*_44_, *s*_*pq*_), clamped ion (*e*_*ik*_^0^), internal strain (*e*_*ik*_^int^), final piezoelectric constant *e*_*ik*_ and *d*_*ik*_ of these materials, and the ZIFs in this work.

**Table 1:** Comparison of Piezoelectric Constants *e*_*ik*_, Its Components *e*_*ik*_^0^ and *e*_*ik*_^int^, *d*_*ik*_ and Compliance Constants *s*_44_ and *s*_*pq*_ of Some Inorganic and Organic Piezoelectrics with ZIFs

properties	ZnX (X = O, S)^68,69^	ZnX (X = Se, Te)^68,69^	CdY (Y = S, Se, Te)^68,69^	PVDF^72^	PVDF–TrFE^72^	ZIFs
|*e*_*ik*_^0^| (C/m^2^)	0.22 to 0.59					≤0.06
|*e*_*ik*_^int^| (C/m^2^)	0.41 to 1.63					≤0.07
|*e*_*ik*_| (C/m^2^)	0.11 to 1.19	0.03, 0.05	0.03 to 0.49	0.01 to 0.27	0.07 to 0.18	≤0.01
*s*_44_ (T Pa^–1^)	10.42 to 34.48	22.67 to 32.15	50.25 to 75.95	454.45	400	558.72 to 5584.61
*s*_*pq*_ (T Pa^–1^)	4.63 to 23.25	8.5 to 24.0	5.72 to 69.20	94.34 to 227.27	90.90 to 714.28	
|*d*_*ik*_| (pC/N)	1.1 to 12.3	0.9 to 1.1	3.92 to 13.98	1.5 to 38.3	7 to 50	0.12 to 46.33

Comparing the results of *e*_*ik*_^0^ and *e*_*ik*_^int^ for inorganic ZnO/ZnS with ZIFs, we see that inorganics have values almost an order to 2 orders of magnitude higher than ZIFs. However, one of the factors that influence *e*_*ik*_^int^ is BECs and BECs of metal *Z*_Zn/Cd_^*^ in ZIFs is similar to BECs of Zn and Cd in the inorganics presented in Table 1. This value is around +2.10 to +2.12 for Zn and + 2.13 to +2.27 for Cd in the inorganics mentioned here, and it is comparable to the values of +2.0 to +2.5 for the ZIFs. The low BECs for Zn^2+^ and Cd^2+^ in both ZnX and CdY ZIFs could be due to the completely occupied d orbitals, unlike Ti^4+^ in BaTiO_3_ with anomalous BEC which has unoccupied d orbitals.^41^ To further understand the differences in *e*_*ik*_^0^ and *e*_*ik*_^int^ between ZnO/ZnS and ZIFs, we looked at the extent of clamped ion contribution that is compensated by internal strain using the ratio −*e*_*ik*_^0^/*e*_*ik*_^int^. For ZnO and ZnS, this is 28–40% and 69–83%, respectively, whereas for all ZIFs, it is 80–100%. Higher compensation in ZnS than ZnO is attributed to the bigger electronic polarizability of sulfide than the oxide anion.^40^ However, for ZIFs in this work, the clamped ion contribution is much smaller than that of inorganics (∼0.06 C/m^2^(ZIFs) versus ∼0.59 C/m^2^(ZnX)), so there is less to compensate for by the internal strain contribution. Plausible reasons for the higher compensation of clamped ion contribution are that (a) ZIFs belong to a nonpolar cubic space group and are highly symmetric and (b) their framework has higher flexibility, by which atoms relax easily in a manner to counter the polarization created via clamped ion contribution. Finally, comparing the |*e*_14_| of all ZIFs which is 0.01 C/m^2^ while that of Zn/Cd inorganic piezoelectrics has values between 0.03 and 1.19 C/m^2^ indicates a low piezoelectric constant “*e*”. Looking at the mechanical properties, the magnitude of *s*_44_ for all ZIFs in this study is greater than ∼560 TPa^–1^ and the highest *s*_44_ among them is for CdIF-1 with a magnitude of ∼5500 TPa^–1^. Between Zn/Cd inorganics and ZIFs, we see that the compliance constant *s*_*pq*_ is very low for inorganics by almost one to 2 orders of magnitude than ZIFs. One thing commonly observed in both Zn/Cd inorganics and Zn/Cd–ZIFs is an increase in *s*_*pq*_ or an improvement in flexibility with a change in metal from Zn to Cd.

Since data for *e*_*ik*_^0^ and *e*_*ik*_^int^ are not available in the literature for organic piezoelectrics PVDF and PVDF–trifluoroethylene (P(VDF–TrFE)), we compare here the BECs (Z*), one of the key contributors to *e*_*ik*_^int^ in these materials with ZIF charges. Previous work on the ferroelectric phase β-PVDF shows that the Z* for C is −0.2/1.45 and that for F and H is −0.75 and 0.14, respectively.^73^ Recent work on metal-free organic perovskite materials such as MDABCO–NH_4_–X_3_ (X = Cl, Br, I) shows C, N, and H with a magnitude of Z* around 0.14 to 0.67.^11^ These values are similar to BECs of the atoms in the organic linkers for ZIFs. Comparing |*e*_14_| of all ZIFs with PVDF and its copolymers, some *e*_*ik*_ values are also as low as ZIFs, but few *e*_*ik*_ values are up to an order of magnitude larger than ZIFs. Also, these organic piezoelectrics are polar materials; hence, a spontaneous polarization occurs by virtue of structure. Mechanical properties of organic materials considered here are lower than those of most ZIFs in some directions, but in a few directions, *s*_*pq*_ is comparable with *s*_44_ of ZIFs (except for CdIF-1 for which *s*_44_ is 1 order of magnitude higher). The literature on clamped ion (*e*_*ik*_^0^) and internal strain contributions (*e*_*ik*_^int^) of *e* for Zn/Cd Se, Te, and polymer piezoelectrics considered here is not available, and these values are not included in Table 1.^a^

Upon the overall comparison of ZIFs with Zn/Cd-based piezoelectric inorganics and specified organics, even though the piezoelectric constant *e* of ZIFs is lower, they are softer and flexible, which is the main contributing factor for obtaining a high piezoelectric coefficient *d*. This is observed in the case of CdIF-1, where |*e*_14_| = ∼0.01 C/m^2^, which is lower than that of most materials shown in Table 1, but it has a |*d*_14_| of 38 to 46 pC/N, which is higher than that of all II–VI-type inorganics and is in the same range of value as the polymer PVDF. After CdIF-1, ZIF-8 with |*d*_14_| = 9 to 14 pC/N is comparable to P(VDF–TrFE) and some Cd inorganics.

Comparing ZIFs with piezoelectrics other than Zn/Cd inorganics and PVDF, ZIF-8 has a *d*_14_ comparable to *d*_33_ of LiNbO_3_ (11 pC/N), poly-l-lactic acid (PLLA) (11 pC/N), and metal-free organic piezoelectric MDABCO–NH_4_I_3_ (14 pC/N).^74^ Piezoelectrics such as PZT (410 pC/N), BaTiO_3_ (191 pC/N), and KNN (80–160 pC/N) (potassium sodium niobate) and recent hybrid materials such as TMCM–MnCl_3_ (185 pC/N) (trimethylchloromethyl ammonium trichloromanganese) have a large piezoelectric response^3,74^ than CdIF-1 in this work. Specifically, for the metal-free MDABCO–NH_4_X_3_ (X = Cl, Br, or I),^11^ the *d*_*ik*_ (250 pC/N) of MDABCO–NH_4_X_3_ is larger than that of all the ZIFs investigated in this work, which is mainly due to larger *e*_*ik*_ (0.35 C/m^2^) than ZIFs because the highest *s*_*pq*_ (650 TPa^–1^) is lower than the *s*_44_ of ZIFs.

The study of cadmium ZIFs in this work provides a good understanding of how flexibility can influence the piezoelectric properties. However, Cd is also a heavy metal like lead, which is toxic and carcinogenic for the human body. Hence, Cd–ZIFs are a proof of concept to show the potential of MOFs as piezoelectric materials but are not ideal candidates for future applications.

### 5.2 Acoustic Properties

Another factor on which the efficiency of piezoelectric devices depends is the proper matching of acoustic impedance (*z*) of energy harvesting materials and the media in which energy is being harvested. Hence, we discuss in brief the acoustic impedance of the ZIFs in this work and compare it with that of some piezoelectric ceramics and polymers and also the acoustic impedance of typical harvesting media. Acoustic impedance is a measure of the ease with which sound travels through a particular medium. It can be calculated as the product of acoustic velocity and density of the material. Theoretically calculated maximum longitudinal acoustic impedance values of ZIFs are shown in Table 2, and detailed maximum and minimum values of longitudinal and transverse velocities are provided in the Supporting Information in Table S3.

**Table 2:** Comparison of Acoustic Impedance (*z*) of ZIFs in This Work with That of Piezoelectric Ceramics, Organics, and Some Energy Harvesting Media

material	acoustic impedance *z*(MRayl)
ZIFs	
ZIF-8	3.10
CdIF-1	2.40
ZIF-90	3.24
ZIF-Cl	3.38
ZIF-65	4.10
CdIF-8	3.37
ceramics^5^	
BaTiO_3_	30.00
PZT4	36.15
LiNbO_3_	34.00
polymers^5^	
PVDF	20
P(VDF–TrFE)	4.51
harvesting media^5^	
water	1.45–1.5
tissue(skin)	1.99

The acoustic impedance values of ZIFs are lower than those of both piezoelectric ceramics and organics. Among the ZIFs, CdIF-1 specifically has a lower acoustic impedance than other ZIFs. Importantly, the acoustic impedance of ZIFs is much closer to harvesting media like water and living tissues, compared to ceramics and some of the polymers. This is an advantage for harvesting energy from these media.

## 6 Conclusions

To obtain a structure–property relationship in MOFs, we systematically investigated the piezoelectric and mechanical properties of six non-centrosymmetric, nonpolar ZIFs with Zn/Cd as metal nodes and varying the substituent on the imidazolate linker (R = CH_3_, Cl, CHO, NO_2_) using DFT calculations. Piezoelectric coefficient *e* is similar in magnitude for all ZIFs because of the huge compensation of clamped ion contribution *e*_14_^0^ by internal strain *e*_14_^int^, possibly from the higher flexibility of the frameworks. The magnitude of “*e*” for the current ZIFs ∼0.01 C/m^2^ is low compared to most inorganic piezoelectrics. This can be tuned for future MOFs by (1) choosing polar MOFs with a spontaneous polarization. Some examples of polar MOFs include Zn–isonicotinate (Zn–IN) MOFs and Zn/Cd/Co-pyridylacrylate MOFs (Zn/Cd/Co-PAA) and (2) MOFs with metal nodes which can show anomalous BECs (Z*) similar to best-performing inorganics BaTiO_3_.

Even though the *e* values for current ZIFs are low, the highest magnitude of piezoelectric constant *d* is seen for CdIF-1 with a Cd metal node and methyl (CH_3_) imidazolate as a linker with a theoretical value of 38–46 pC/N. This value is comparable to those of the most common organic piezoelectric PVDF, and the main contributing factor for this high *d* value is the low shear modulus (high flexibility) of the framework. The estimated FOM of CdIF-1 calculated using an experimentally measured dielectric constant value of ZIF-8 (2.33) and *d* values obtained in this work is 620/ε_0_ to 900/ε_0_ (pC/N)^2^. This is quite high compared to the FOM_*ij*_ of PZT (410 pC/N, ε = 1800) and PVDF (30 pC/N, ε = 10),^75^ 93/ε_0_ and 90/ε_0_ (pC/N)^2^, respectively. Hence, when tunable parameters such as structural building units are optimized to obtain high flexibility, high BECs, and favorable non-centrosymmetric symmetry, MOFs should be exceptional candidates to be used as piezoelectric energy harvesters.

## Supporting Information Available

Detailed theory of piezoelectricity; geometry optimization details and lattice parameters; computed piezoelectric constants *e*_14_, *d*_14_, shear modulus *c*_44_, compliance constant *s*_44_ for all six ZIF structures with five different DFT functionals; and different parts of internal strain contribution *e*_14_^int^, that is, change in structural parameters and BECs for all ZIFs (PDF)
Piezoelectric and mechanical properties results (XLSX)
Atomwise internal strain using B3LYP (XLSX)
Representative input files of calculations done using CRYSTAL17 (ZIP)
Representative output files of calculations done using CRYSTAL17 (ZIP)

## Notes

The raw data files with the representative input files and output files of the calculations done in this work can also be accessed by others at 4TU.ResearchData: https://doi.org/10.4121/19168730.v2.

## Footnotes

a II–VI-type inorganics with Zn and Cd such as ZnO and ZnS exist either in the hexagonal wurtzite phase or cubic zinc blende phase and have 3 (*e*_33_, *e*_31_, *e*_15_) and 1 (*e*_14_) independent piezoelectric constant, respectively.
