# Supplementary material for: Structure–Property Relationship of Piezoelectric Properties in Zeolitic Imidazolate Frameworks: A Computational Study
Source: ACS Appl Mater Interfaces. 2022 Nov 2;14(45):50803–14. doi: 10.1021/acsami.2c13506 (PMC9674201; doi:10.1021/acsami.2c13506)
Supplement: Supplementary file 1 — am2c13506_si_001.pdf [file am2c13506_si_001.pdf]

Supporting Information

Structure-Property Relationship of Piezoelectric Properties in Zeolitic Imidazolate Frameworks (ZIFs): A Computational Study

Srinidhi Mula,† Lorenzo Donà,‡ Bartolomeo Civalleri,*,‡ and Monique A. van der Veen*,†

†*Department of Chemical Engineering, Technische Universiteit Delft, Delft, 2629HZ, The Netherlands*

‡*Department of Chemistry, Universita degli Studi di Torino, Torino, 10124, Italy*

E-mail: bartolomeo.civallleri@unito.it; m.a.vanderveen@tudelft.nl

1 Theory

Piezoelectricity is mathematically described in the IEEE standard for piezoelectricity [1] by a set of four constitutive equations that describe the response of a piezoelectric material to a mechanical load (stress/strain) and electric fields. Of relevance in this work are a set of constitutive equations shown in equation 1.

$$\begin{aligned} D_i &= e_{ikl}S_{kl} + \varepsilon^S_{ij}E_k \\ S_{ij} &= s^E_{ijkl}T_{kl} + d_{kij}E_k \end{aligned} \tag{1}$$

where D, S, ε, E, s, T represent electric displacement [C/m^2] strain tensor (dimensionless), dielectric constant [F/m], electric field [V/m], elastic compliance tensor [m^2/N] and stress tensor [N/m^2] respectively. e and d are piezoelectric tensors with units [C/m^2] and [pC/N]

respectively. Note that of importance in piezoelectric applications is the value of d which effectively links the applied electrical field to the deformation of the material (strain S), relevant for transducers, or the applied stress (T) to the electric displacement (D) which can be stored as energy in piezoelectric energy harvesting. This relation between electrical and mechanical properties is pictorially shown by Heckmann diagram in Figure S1 adapted from the book by JF Nye.[2]

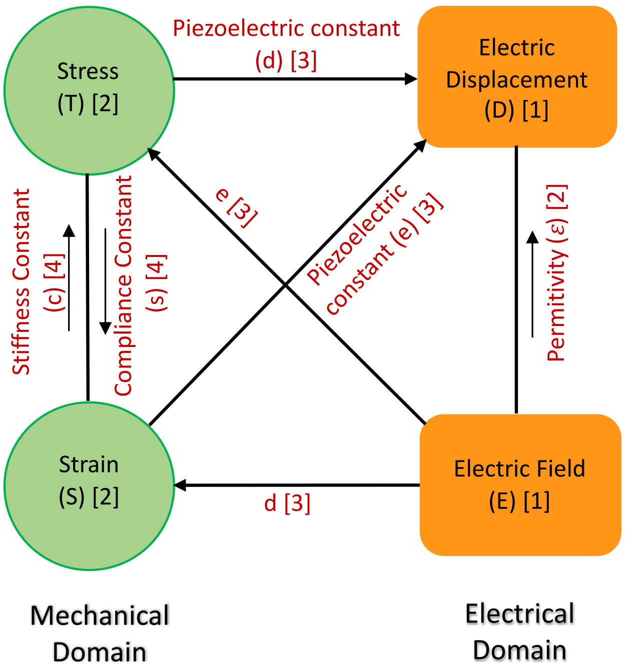

Figure S1: Part of Heckmann diagram showing the relation between mechanical and electrical properties, various variables, and their symbols. The tensor rank of the properties and variables is shown in square brackets.

We will use Voigt notation for the fourth-order elastic (s^{E}_{ijkl}) and third order piezoelectric tensors (e_{ikl}, d_{kij}) where the indices are given in compressed matrix notation instead of the tensor notation. This means that the indices ij or kl where $i, j, k, l = 1, 2, 3$ are replaced by p or q and pairs of contracted cartesian directions $p, q = 1, 2, 3, 4, 5, 6$ where 11:1; 22:2; 33:3; 23, 32:4; 13, 31:5; 12, 21:6. The piezoelectric constant d_{ip} can be computed from the piezoelectric constant e and the elastic compliance constant s using $d_{ip} = e_{iq}s^{\mathrm{E}}_{qp}$. Computationally, the piezoelectric constant e_{iq} is calculated as the first derivative of the magnitude

of the polarization P induced by strain $e_{iq} = \left(\frac{\partial P_i}{\partial \varepsilon_q}\right)_E$.

2 Geometry Optimization

Experimental structures deposited in CSD database[3] were used as starting geometry for full relaxation of ZIF structures except for ZIF-90 for which the structure was obtained from single crystal XRD measurements of synthesized crystals in the group. We adopted a series of five different functionals (B3LYP-D3, PBE0-D3, M06-D3, M06L-D3 & M062X) and with all functionals geometry optimization, vibrational frequency calculation followed by a piezoelectric calculation was done. Clamped Ion contribution to piezoelectric constant was calculated in a separate calculation. Vibrational frequency calculation was done to ensure the equilibrium structure is at the minima of the potential energy surface.

Note on Methyl ZIFs: ZIF-8 and CdIF-1 belong to $I\bar{4}3m$ space group where the hydrogens of methyl group are disordered due to free rotation of methyl groups.[4] Hence, for modelling purposes, the symmetry was lowered to $I23$ where orientation of methyl group can be present in two orientations as shown in Figure S2. For each DFT functional, we compared the final energies of the two equilibrium structures, the vibrational frequencies and convergence of the piezoelectric calculations for the structures. Piezoelectric constants of the structure with the lowest energy, absence of negative frequencies and converged piezoelectric calculation was considered for each functional and shown in the next sections of SI. For ZIF-8, the orientation of methyl groups shown on the left in Figure S2 was considered final for all DFT functionals except for M062X functional. With M062X functional, the methyl orientation on the right in Figure S2 had the lowest energy, and the piezoelectric calculation converged well for this structure. In case of CdIF-1, with B3LYP-D3 and PBE0-D3 orientation shown on left in Figure S2 was used and with M06L-D3 and M06-D3 functionals, structure on the left was considered for comparison of final piezoelectric and mechanical properties. For CdIF-1, the piezoelectric calculations did not converge with M062X DFT functional for both

orientations even with stringent convergence criteria. Hence, this result was not considered for comparisons and marked separately in all graphs in the next sections of SI.

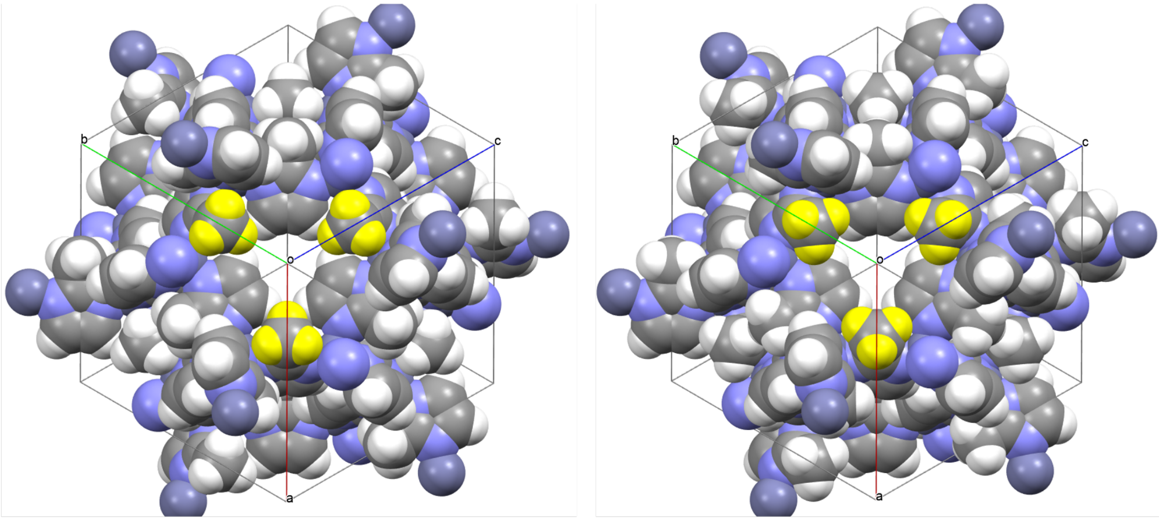

Figure S2: Two possible orientations of Methyl groups highlighted for the case of ZIF-8.

Note on ZIF-90: The experimental structure of ZIF-90 published in the CSD database belongs to space group $I\bar{4}3m$ and oxygen, hydrogen of the aldehyde group are disordered due to symmetry.[5] Hence, for modelling, the symmetry was reduced to I23. On geometry optimization of this lower symmetry structure, it converged to a structure similar to the single crystal XRD structure we obtained from synthesis in the group. Hence, the unpublished single crystal XRD structure was used as the starting structure for all further piezoelectric calculations.

The simulated parameters of final optimized structures and deviation from experimental parameters for each DFT functional is shown in Table S1.

Table S1: Experimental and Optimized lattice parameter 'a' along with deviation (Δ%) for all five different DFT functionals in each ZIF

DFT Functional	Lattice Parameter	ZIF-8	CdIF-1	ZIF-90	ZIF-Cl	ZIF-65	CdIF-8
Experimental	a (Å)	17.009[6]	18.121[7]	17.060[§]	17.037[8]	17.000[9]	18.077[7]
M06L-D3	a (Å)	16.877	17.953	17.148	17.041	17.152	18.035
	Δ(%)	-0.78	-0.92	0.52	0.03	0.90	-0.23
B3LYP-D3	a (Å)	17.078	18.126	17.261	17.116	17.294	18.127
	Δ(%)	0.40	0.03	1.18	0.46	1.73	0.27
PBE0-D3	a (Å)	17.007	18.037	17.043	17.053	17.212	18.045
	Δ(%)	-0.01	-0.46	-0.10	0.09	1.25	-0.18
M06-D3	a (Å)	16.819	17.870	17.077	16.941	17.184	17.962
	Δ(%)	-1.12	-1.39	0.10	-0.56	1.08	-0.64
M062X	a (Å)	16.927	18.045	17.006	17.182	17.212	18.019
	Δ(%)	-0.48	-0.42	-0.32	0.85	1.25	-0.32

3 Variation of Piezoelectric constant 'e' and its components with DFT functional

Variation of piezoelectric constant 'e' is inconsistent across all types of ZIF, meaning the variation with functional is large for some ZIFs, and it is small for others, as shown in Figure S3. In e_{14}^{0} (clamped ion) calculations, we found that the ratio of standard deviation (SD) to average e_{14}^{0} with DFT functionals ranges between 1 – 14% for all ZIFs. This is the same for e_{14}^{int} (internal strain) except for ZIF-90 and CdIF-8 a large deviation of 24% and 27% is observed. Similar variation of piezoelectric constants of about 40% was observed in inorganics based on the choice of LDA or GGA approximation to exchange-correlation energy, even though change in lattice constants is 1-2%.[10] In the case of ZIF8, CdIF-1 and ZIF-65 for all DFT functionals, the sign of e_{14} follows the sign of e_{14}^{int} indicating $e_{14}^{\text{int}} > e_{14}^{0}$ i.e., internal strain values are larger than the clamped ion values for these ZIFs. For CdIF-8, we see that for all functionals $e_{14}^{\text{int}} < e_{14}^{0}$, thus e_{14} has the same sign as e_{14}^{0}. For ZIF-90 and ZIF-Cl

[§]Structure taken from unpublished single crystal XRD data with a spacegroup of I23

the sign of e_{14} differs based on the choice of functional. In the case of B3LYP and PBE0 for ZIF-Cl $e_{14}^{\text{int}} = e_{14}^{0}$, so e_{14} is zero. In Figure S3, internal strain e_{14}^{int} and e_{14} are marked in the graph for CdIF-1 with M062X functional and for ZIF-65 with M06-D3 functional. These specific piezoelectric calculations did not converge properly even with stringent convergence criteria on energy, hence these results were excluded for conclusions obtained in the main paper.

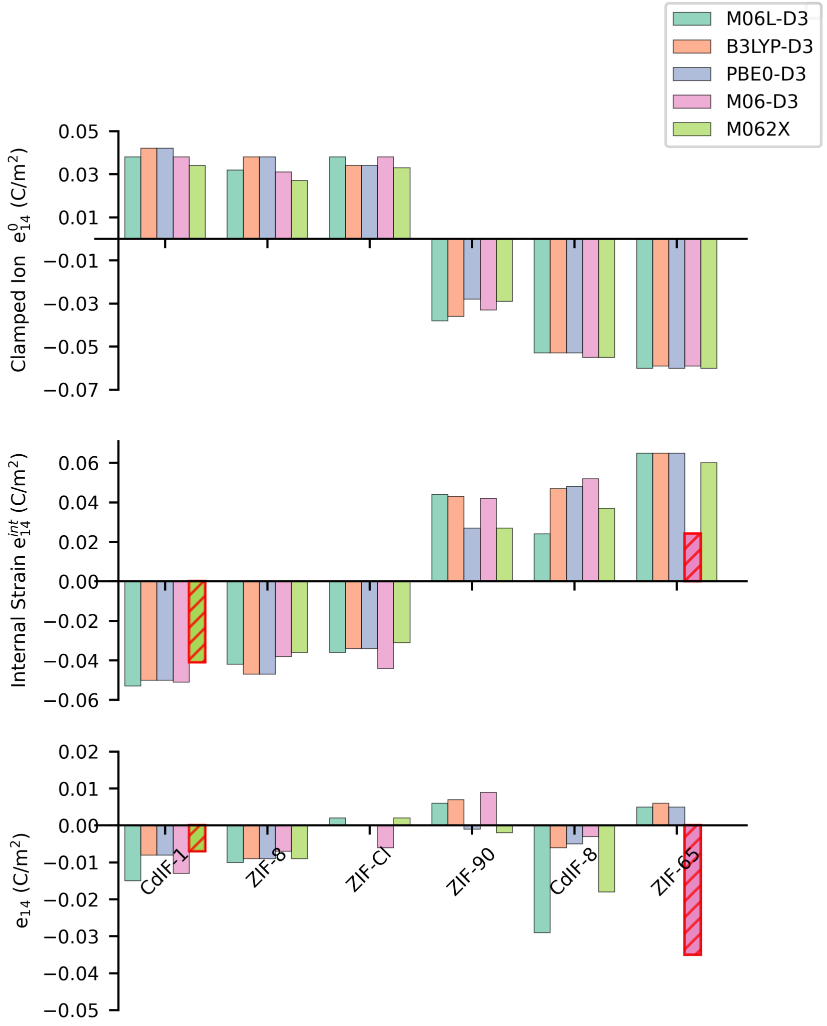

Figure S3: Clamped ion e_{14}^{0}, Internal Strain contributions e_{14}^{int} and total piezoelectric constant e_{14} with five different DFT functionals for the considered ZIFs.

4 Different parts of Internal Strain Contribution e_{14}^{int}

4.1 Bond lengths

For all ZIFs we see that the highest change in bond length occurs in the Metal-N bonds, ranging from 0.24% for ZIF-65 to 0.06% for ZIF8 (see Table S2). In methyl ZIFs (ZIF-8 and CdIF-1), the second-largest change in bond length was in the C-N bonds of the imidazolate ring, with the C bonded to the methyl substituent. In the other 4 ZIFs, substituted with -Cl, CHO and -NO_2, we find that apart from the Metal–N bonds, the largest changes in bond length occur in a) the carbon–substituent bonds, b) bonds within the substituent itself and c) for ZIF-90 and ZIF-65, the C–N bond within the imidazolate ring with C not bonded to the substituent , and for ZIF-Cl and CdIF-8 the C–N bond with C bonded to the substituent.

4.2 Born effective Charges (Z^*)

The labelling of the different atom types in the organic linker can be found in Figure S4. Born effective charges Z^* of all the atoms in the organic linker C1, C2/C3,N, H and X are shown in Figure S5. Within the imidazolate ring, the Z^* values vary between ± 1 for C1, ± 0.6 for C2/C3 and -1.00 to $+\,0.75$ for N. This varied distribution of charges in the atoms of the linkers ranging from +1 to -1 for different types of C and N atoms indicates a covalent nature of bonds between them. Unlike non-substituent linker atoms, the BEC of different elements in the substituent (X) for each ZIF show distinct values, as seen in Figure S5(e). In ZIF-8 and CdIF-1 i.e. -CH_3 ZIFs, all BEC's of C and H are in ± 0.15. For ZIF-Cl, all Cl atoms have BECs in $+0.25$ to $-\,0.4$, in ZIF-90, ZIF-65 and CdIF-8 most BEC values of C, H, N, O fall in the range -0.8 to $+\,1.0$. In ZIF-90, the BEC value of C and O of some linkers is higher than other linkers with a value as high as ± 1.5. A similar trend is seen in ZIF-65 and CdIF-8 with N and O having higher BEC values of $+2.2$ and $-\,1.6$ respectively.

Table S2: Variation due to external shear in bond lengths and bond angles above for all ZIF structures. A cutoff of 0.05% for bond lengths and 1° change for bond angles was chosen and values higher are shown in the table

MOF	Atoms Involved	Relative Bond length change (%)	Atoms Involved	Bond angle change (°)
ZIF-8	Zn-N	0.06	N-Zn-N	3.12
	C-N (where C is bonded to $-CH_3$ substituent)	0.04	Zn-N-C	0.89
CdIF-1	Cd-N	0.10	N-Cd-N	3.41
	C-N (where C is bonded to $-CH_3$ substituent)	0.03	Cd-N-C	0.80
ZIF-90	Zn-N	0.14	N-Zn-N	3.48
	C-C (where C is bonded to -CHO substituent)	0.10	Zn-N-C	1.44
	C-O (Bond in the -CHO substituent)	0.08		
	C-H (Bond in the -CHO substituent)	0.08		
	C-N (where C is not bonded to -CHO substituent)	0.07		
ZIF-Cl	Zn-N	0.06	N-Zn-N	3.27
	C-N (where C is bonded to -Cl substituent)	0.05	Zn-N-C	0.69
	Cl-C (where C is bonded to -Cl substituent)	0.05		
ZIF-65	Zn-N	0.24	N-Zn-N	2.90
	C-N (where C is bonded to $-NO_2$ substituent)	0.09	Zn-N-C	1.18
	C-N (where C is not bonded to $-NO_2$ substituent)	0.08		
	N-O (Bond in the $-NO_2$ substituent)	0.07		
CdIF-8	Cd-N	0.10	N-Cd-N	3.23
	N-O (Bond in the $-NO_2$ substituent)	0.06	Cd-N-C	1.13
	C-N (where C is bonded to $-NO_2$ substituent)	0.04		

4.3 Combined contribution of Born effective charges and relaxation coefficients 'A' $= \left(Z^*_{s,1j} * \frac{\partial x_{sj}}{\partial \varepsilon_4}\right)$ to e^{int}_{14}

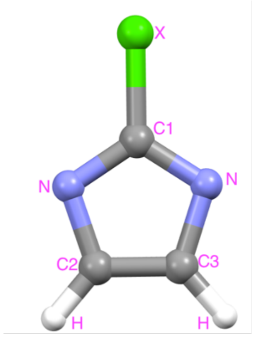

Figure S4: Atoms in the organic unit labelled according to the asymmetric unit of the ZIF system

As e^{int}_{14} is defined by the product of the BEC's and relaxation coefficients (equation 5 in main paper), we identified the atoms which have the highest magnitude for this product 'A' $= \left(Z^*_{s,1j} * \frac{\partial x_{sj}}{\partial \varepsilon_4}\right)$ to e^{int}_{14} in all ZIFs. For each ZIF, a cutoff value of 5% of $|e^{\text{int}}_{14}|$ was chosen to consider the product 'A' $= \left|Z^*_{s,1j} * \frac{\partial x_{sj}}{\partial \varepsilon_4}\right|$ for all atoms. All atoms with absolute contributions greater than this value are

highlighted in Figure S6, where green indicates a positive contribution and red indicates a negative contribution to internal strain contribution e_{14}^{int}. In ZIF-Cl (Figure S6(b)), C1, C2/C3 and H atoms of the imidazolate have product greater than cutoff and metal atoms have low contribution than the cutoff. However, in ZIF-90, ZIF-65 and CdIF-8, (Figure S6(a, c, d)) metal atoms (Cd or Zn) have both positive and negative product 'A'> cutoff. In the organic part for these 3 ZIFs, C1, some C2/C3 carbons, some N atoms, and atoms in the substituent groups i.e., C, O of -CHO and N, O of -NO_2 all of them have higher contributions than the cutoff. For the same cutoff on the product 'A' for all ZIFs, a clear difference in the number of atoms that have 'A' > cutoff is seen in ZIF-90, Nitro ZIFs vs methyl ZIFs and ZIF-Cl.

5 Variation of Mechanical Properties and Piezoelectric constant 'd' with DFT functional

In Figures S7a, S7b, S8, S9 and S10, c_{44}, s_{44}, bulk properties and d_{14} are marked in the graph for CdIF-1 with M062X functional and for ZIF-65 with M06-D3 functional. These specific piezoelectric calculations did not converge properly even with stringent convergence criteria on energy, hence these results were excluded for conclusions obtained in the main paper.

5.1 Shear constant c_{44} and Compliance constant s_{44}

For mechanical properties c_{44} and s_{44}, the ratio of the standard deviation to the average varies from 0.1 – 18% except for CdIF-1 and CdIF-8 a deviation of 30% is observed. Although c_{44} and s_{44} vary with the choice of DFT functionals for each ZIF, variation between different ZIFs is consistent in most cases.

5.2 Bulk properties

From the complete elastic tensor obtained in the calculations, mechanical properties like Young's modulus, Bulk modulus and Poisson ratio are also computed using Voigt-Reuss-Hill(VRH) approximation. Hence, we show in Figure S8 these properties for all ZIFs calculated with different DFT functionals. Bulk moduli for all ZIFs in this work are less than 13 GPa. They are much smaller than Bulk moduli of inorganic perovskite $BaTiO_3$ (162 GPa), $PbTiO_3$ (144 GPa).[11] Young's moduli of ZIFs are all below 5 GPa which are the same order of magnitude as PVDF (4.18 GPa) and PVDF-TrFE (1.5 GPa), but lower than piezoelectric ceramics like PZT and $BaTiO_3$.[12]

5.3 Piezoelectric constant 'd'

As shown in Figure S10, there is a lot of variation of d_{14} with the choice of DFT functional, due to the variability of both e_{14} (Figure S3) and s_{44} (Figure S7b) with choice of functional.

6 Acoustic Properties of ZIFs

The acoustic velocities are obtained as a part of the calculations, by solving the Christoffel equations based on the elastic constants and densities.

Table S3: Acoustic properties of all six ZIFs

Properties	ZIF-8	CdIF-1	ZIF-90	ZIF-Cl	ZIF-65	CdIF-8
Density (kg/m^3)	904	923	984	1057	1109	1131
Minimum velocities (km/s)						
Longitudinal	3.373	2.524	3.151	3.140	3.590	2.957
Transverse (v_1)	0.920	0.442	1.011	0.929	1.102	0.810
Transverse (v_2)	0.920	0.442	1.011	0.929	1.008	0.787
Maximum velocities (km/s)						
Longitudinal	3.427	2.602	3.289	3.198	3.699	2.982
Transverse (v_1)	1.015	0.705	1.298	1.066	1.270	0.854
Transverse (v_2)	1.015	0.629	1.210	1.022	1.270	0.854
Acoustic Impedance (Longitudinal)(MRayl)						
Minimum	3.05	2.33	3.10	3.32	3.98	3.34
Maximum	3.10	2.40	3.24	3.38	4.10	3.37

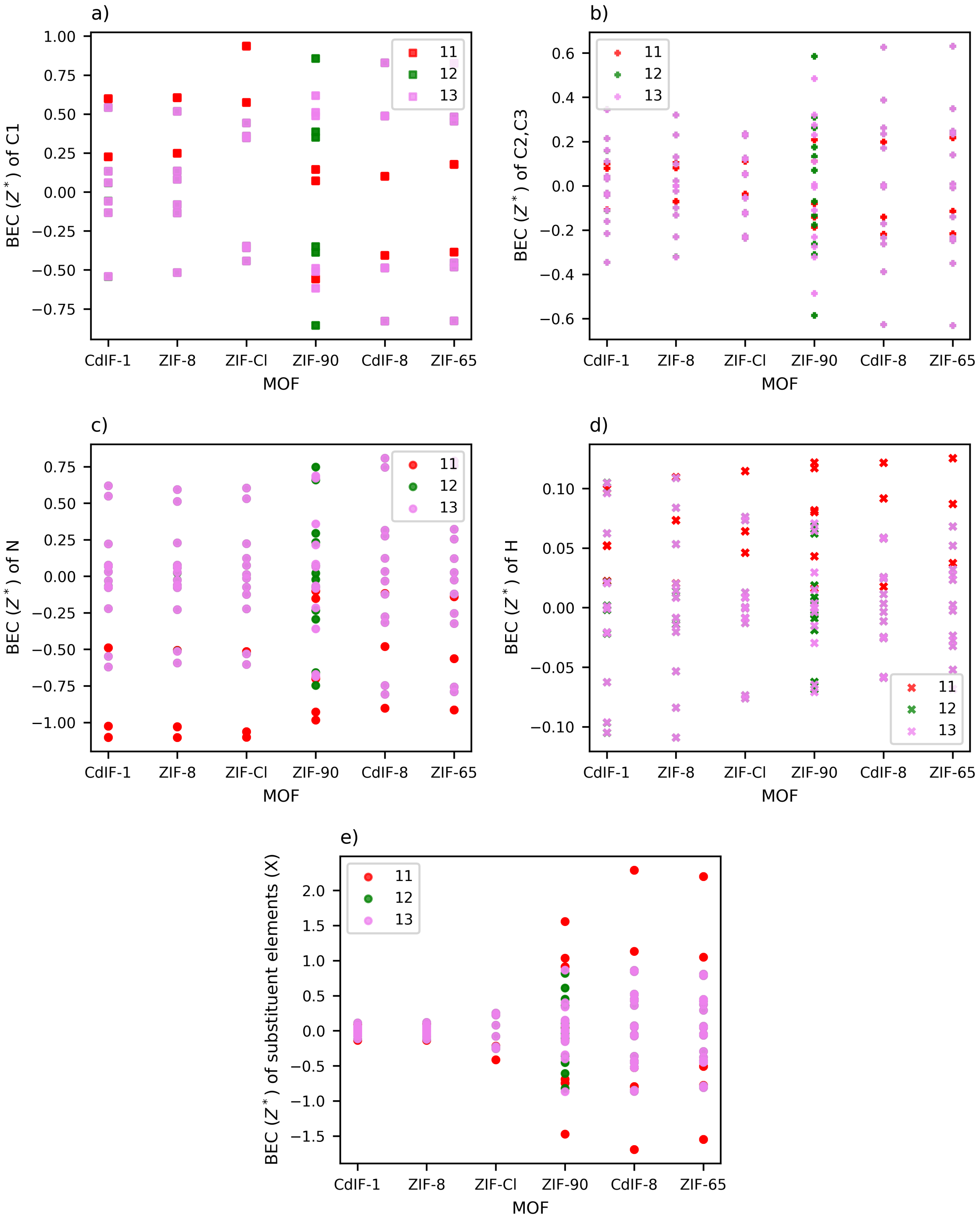

Figure S5: Born effective charges Z^* in relevant directions (11,12,13) for a) C1 carbon b) C2/C3 carbon c) N atoms d) H atoms e) substituent atoms in X

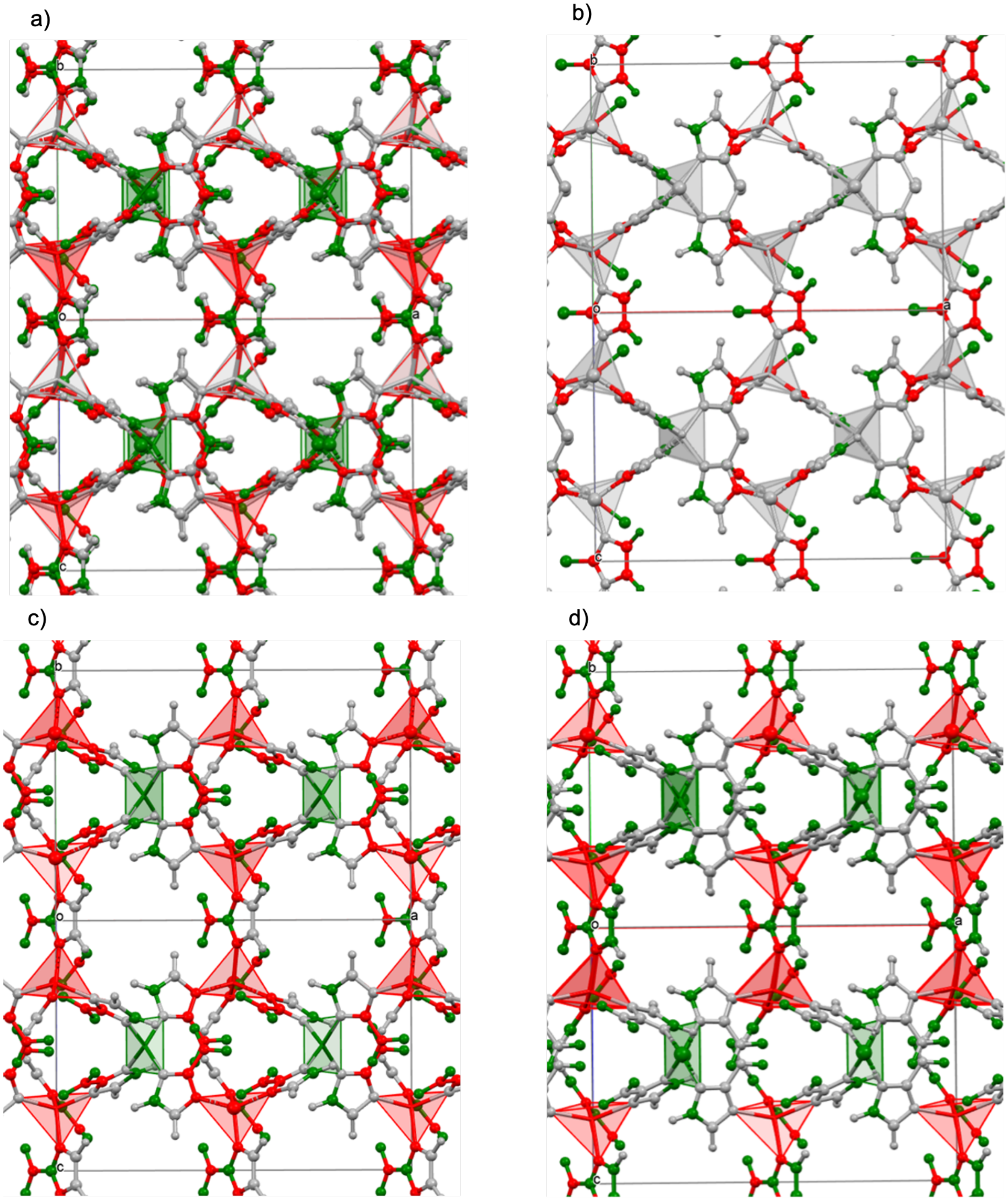

Figure S6: ZIF structures highlighting the atoms with contribution 'A' $= \left| Z^{*}_{s,1j} * \dfrac{\partial x_{sj}}{\partial \varepsilon_4} \right|$ greater than 5% of e^{int}_{14} a) ZIF-90 b) ZIF-Cl c) ZIF-65 and d) CdIF-8. The positive contribution of 'A'is indicated with green color and negative contribution in red colour.

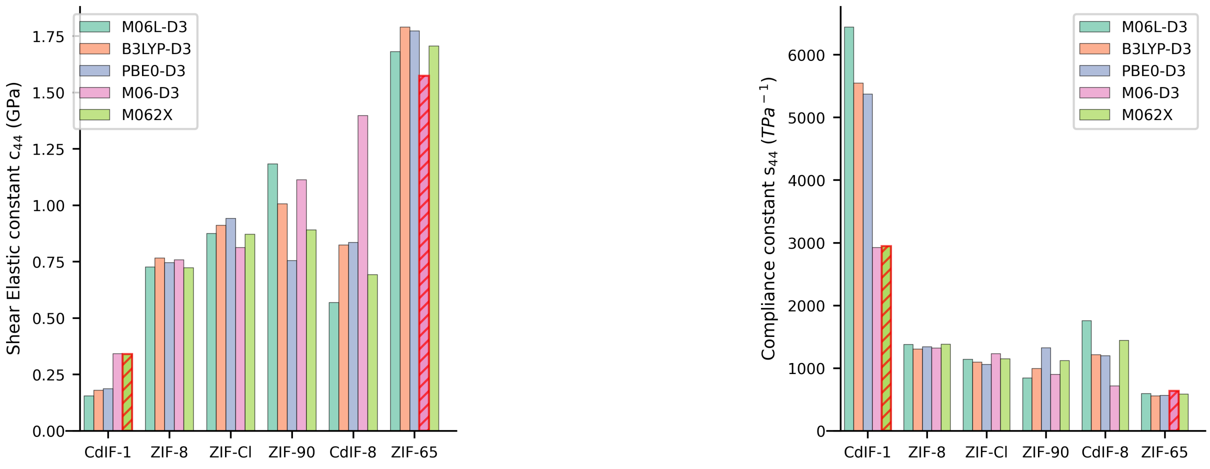

(a) (b)

Figure S7: Bar plots of (a)Shear elastic constant (c_{44})and (b) Compliance constant (s_{44}) for all ZIFs with five DFT functionals

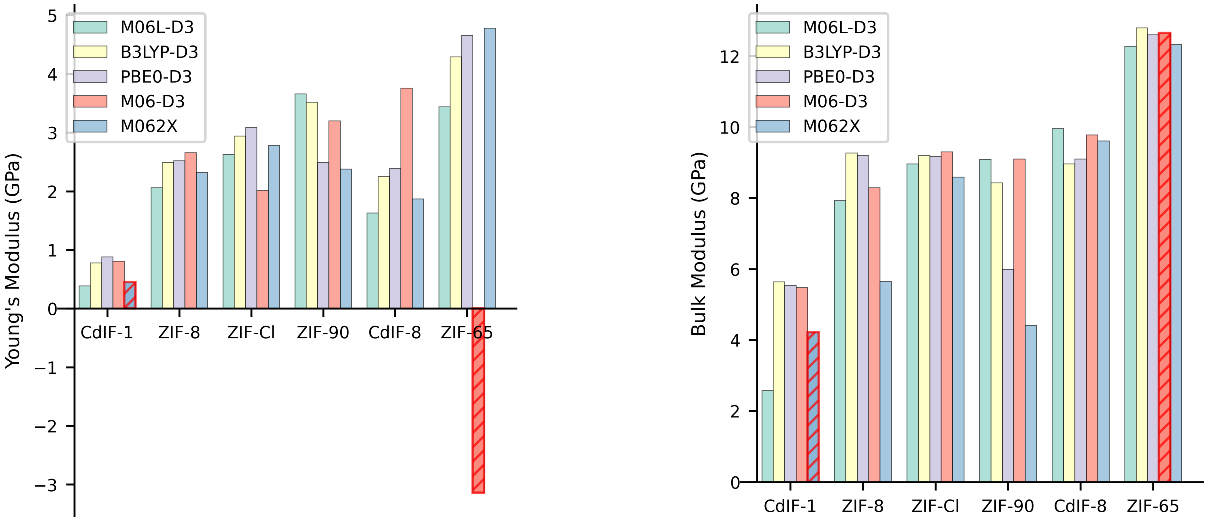

(a) (b)

Figure S8: Bar plots of (a)Young's Modulus and (b) Bulk Modulus for all ZIFs with five DFT functionals

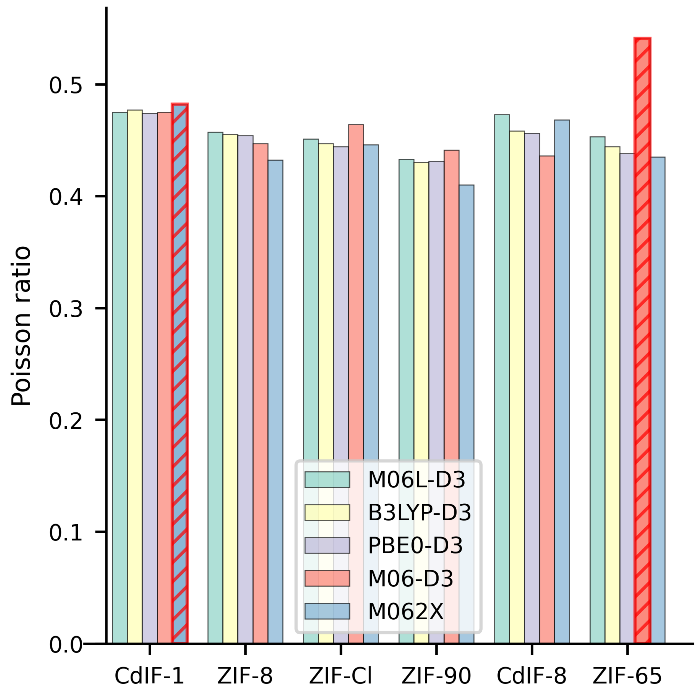

Figure S9: Poisson's ratio, obtained with five different DFT functionals for all ZIFs

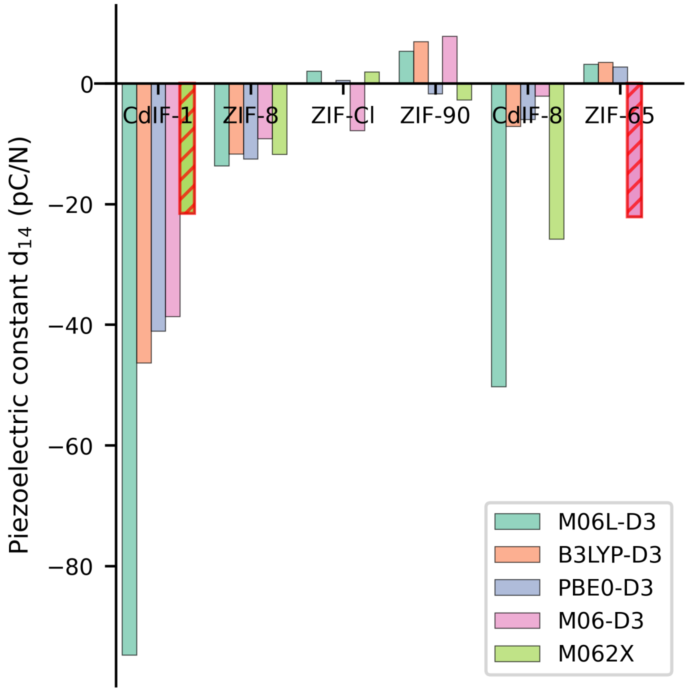

Figure S10: Piezoelectric constant d_{14} obtained with five different DFT functionals for all ZIFs

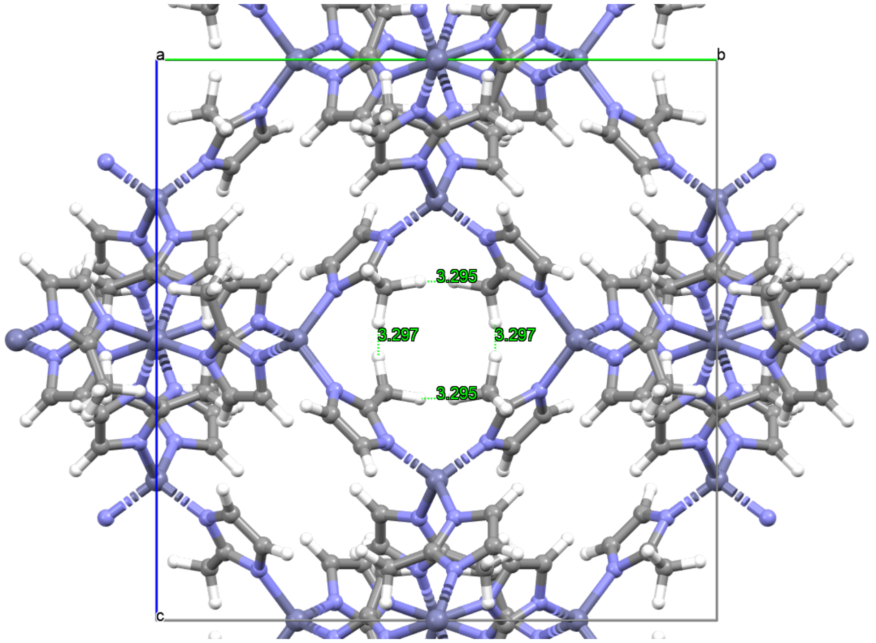

(a)

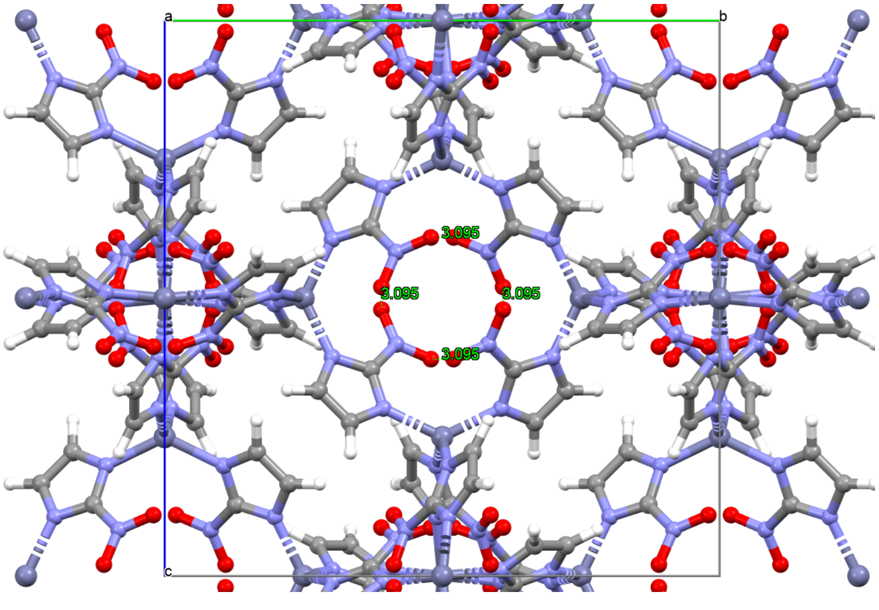

(b)

Figure S11: Structures of a) ZIF-8 and b) ZIF-65 showing the (100) plane with the distance between H–H of -CH_3 groups and O–O of -NO_2 groups in the pore indicated.

References

(1) American, A.; Standard, N. IEEE Standard on Piezoelectricity. *ANSI/IEEE Std 176-1987* **1988**, 0_1–.

(2) Nye, J. F. *Physical properties of crystals: their representation by tensors and matrices*; Clarendon Press, 1985: New York, 1985.

(3) Groom, C. R.; Bruno, I. J.; Lightfoot, M. P.; Ward, S. C. The Cambridge Structural Database. *Acta Crystallographica Section B Structural Science, Crystal Engineering and Materials* **2016**, *72*, 171–179.

(4) Li, Q.; Zaczek, A. J.; Korter, T. M.; Zeitler, J. A.; Ruggiero, M. T. Methyl-rotation dynamics in metal–organic frameworks probed with terahertz spectroscopy. *Chemical Communications* **2018**, *54*, 5776–5779.

(5) Morris, W.; Doonan, C. J.; Furukawa, H.; Banerjee, R.; Yaghi, O. M. Crystals as Molecules: Postsynthesis Covalent Functionalization of Zeolitic Imidazolate Frameworks. *Journal of the American Chemical Society* **2008**, *130*, 12626–12627.

(6) Morris, W.; Stevens, C. J.; Taylor, R. E.; Dybowski, C.; Yaghi, O. M.; Garcia-Garibay, M. A. NMR and X-ray Study Revealing the Rigidity of Zeolitic Imidazolate Frameworks. *The Journal of Physical Chemistry C* **2012**, *116*, 13307–13312.

(7) Tian, Y.-Q.; Yao, S.-Y.; Gu, D.; Cui, K.-H.; Guo, D.-W.; Zhang, G.; Chen, Z.-X.; Zhao, D.-Y. Cadmium Imidazolate Frameworks with Polymorphism, High Thermal Stability, and a Large Surface Area. *Chemistry - A European Journal* **2010**, *16*, 1137–1141.

(8) Chaplais, G. G.; Fraux, G.; Paillaud, J.-L.; Marichal, C.; Nouali, H.; Fuchs, A. H.; Coudert, F. O.-X.; Patarin, J. L. Impacts of the Imidazolate Linker Substitution (CH

3 , Cl, or Br) on the Structural and Adsorptive Properties of ZIF-8. *J. Phys. Chem. C* **2018**, *122*, 47.

(9) Diring, S.; Wang, D. O.; Kim, C.; Kondo, M.; Chen, Y.; Kitagawa, S.; Kamei, K.-I.; Furukawa, S. Localized cell stimulation by nitric oxide using a photoactive porous coordination polymer platform. *Nature Communications* **2013**,

(10) de Jong, M.; Chen, W.; Geerlings, H.; Asta, M.; Persson, K. A. A database to enable discovery and design of piezoelectric materials. *Scientific Data* **2015**, *2*, 150053.

(11) Piskunov, S.; Heifets, E.; Eglitis, R.; Borstel, G. Bulk properties and electronic structure of SrTiO3, BaTiO3, PbTiO3 perovskites: an ab initio HF/DFT study. *Computational Materials Science* **2004**, *29*, 165–178.

(12) Habib, M.; Lantgios, I.; Hornbostel, K. A review of ceramic, polymer and composite piezoelectric materials. *Journal of Physics D: Applied Physics* **2022**, *55*, 423002.
